# Azaheterocyclic diphenylmethanol chiral solvating agents for the NMR chiral discrimination of alpha-substituted carboxylic acids†

**DOI:** 10.1039/d0ra06312f

**Published:** 2020-09-18

**Authors:** Gao-Wei Li, Xiao-Juan Wang, Dan-Dan Cui, Yu-Fei Zhang, Rong-Yao Xu, Shuai-Hua Shi, Lan-Tao Liu, Min-Can Wang, Hong-Min Liu, Xin-Xiang Lei

**Affiliations:** College of Chemistry and Chemical Engineering and Henan Engineering Laboratory of Green Synthesis for Pharmaceuticals, Shangqiu Normal University Shangqiu 476000 P. R. China juanzi409e@126.com liult05@iccas.ac.cn; School of Pharmaceutical Sciences, Zhengzhou University Zhengzhou 450001 P. R. China; School of Pharmaceutical Sciences, South-Central University for Nationalities Wuhan 430074 P. R. China xxlei@mail.scuec.edu.cn

## Abstract

A series of small-membered heterocycle probes, so-called azaheterocycle-containing diphenylmethanol chiral solvating agents (CSAs), have been developed for NMR enantiodiscrimination. These chiral sensors were readily synthesized were inexpensive and efficiently used for the chiral analysis of alpha-substituted carboxylic acids. The sensing method was operationally simple and the processing was straightforward. Notably, we propose (*S*)-aziridinyl diphenylmethanol as a promising CSA, which has excellent chiral discriminating properties and offers multiple detectable possibilities pertaining to the ^1^H NMR signals of diagnostic split protons (including 25 examples, up to 0.194 ppm, 77.6 Hz). Its ability to detect the molecular recognition of fluorinated carboxylic acids were further investigated, with a good level of discrimination *via* the ^19^F NMR spectroscopic analysis. In addition, an accurate enantiomeric excess (ee) analysis of the *p*-methoxyl-mandelic acid with different optical compositions have been calculated based on the integration of well-separated proton signals.

## Abstract 2

Chiral azaheterocycle-containing diphenylmethanols with multiple hydrogen-bonding sites were described and used as NMR chiral solvating agents (CSAs). Highly resolved NMR spectra can be obtained directly in the NMR tube.

## Graphical abstract

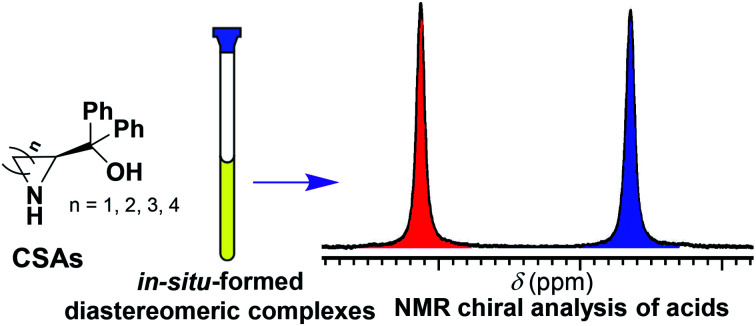

The detection and identification of chiral compounds have attracted considerable scientific attention due to their indispensable role in technological applications including pharmacology, biology, and modern chemistry for the last several decades.^1^ So far, spectroscopic and chromatographic approaches are generally utilized for the enantioselective molecular recognition or quantitative analysis including enantio-discrimination, measurement of enantiomeric excess (ee) and assignment of absolute configuration.^2^ Among them, the nuclear magnetic resonance (NMR) spectroscopy is one of the most convenient, versatile, and routinely available analytical techniques because it provides structural information in a multidimensional manner. For these NMR-based analytical methods, the use of diastereomers prepared from chiral solvating agents (CSAs) and guest compounds has become an efficient and operationally simple analytical approach for chiral discrimination.^3^ Compared with chiral derivatization agents (CDAs), CSAs form two *in situ*-formed diastereomeric complexes with analytes *via* non-covalent interactions, which can be tested immediately after mixing, avoiding chiral derivatization in advance. Over the last few decades, a number of CSAs have been developed, such as coordinative unsaturated chiral lanthanide or transition metal complexes,^4^ low molecular-weight synthetic Brønsted acids/bases,^5^ and supramolecular receptors,^6^ to generate anisochronous chemical shifts. Furthermore, some of them are obtained *via* synthesis and some from nature. It has been reported that enantiopure compounds/complexes are good NMR CSAs for the determination of the enantiomeric purity of different chiral substrates including alcohols or 1,2-diols, cyanohydrins, and hydroxy acids or amino acid derivatives.^7^ Although several CSAs are commercially available, the current methods possess several drawbacks such as line-broadening, narrow substrate scope, poor solubility, and poor resolution. Thus, the development of excellent CSAs with wide applications of the ^1^H NMR chiral analysis is still highly desirable.

Small-membered azaheterocycle derivatives have gained a lot of interest among organic and medicinal chemists for the synthesis of biological active nitrogen-containing compounds. Now, 2-substituted aziridines or azetidines have been already used as synthons for the synthesis of a wide range of compounds in stereoselective transformations, such as highly stereoselective addition or reduction, regioselective ring expansion reaction, and nucleophilic ring-opening polymerization.^8^ Their broad utility has made these aziridine, azetidine, and even pyrrolidine-based more specifically optically pure amino alcohols, a class of useful and attractive chiral ligands/catalysts in a number of catalytic asymmetric transformations, including the addition of transition metal-catalyzed and organocatalytic asymmetric processes.^9^ Among various *N*-heterocyclic amino alcohols, such as *N*-trityl aziridine carbinols,^10^ Ferrocenyl-substituted aziridinylalcohols,^11^*N*-phenylethyl aziridine carbinols,^12^*N*-substituted azetidino alcohols,^13^ and azetidine-2-carboxamides,^14^ diphenylprolinols^15^ have received considerable attention and have been successfully applied to catalyze asymmetric reactions. Nevertheless, the literature concerning the guests of chiral analysis using a small-membered *N*-heterocyclic moiety and the study on enantiodiscrimination in isotropic solutions by NMR techniques are still not abundant. A few representative ones are the following: Rachwalski *et al.* explored chiral aziridine-mediated imines to evaluate their action as CSAs towards mandelic derivatives,^16^ and proline-derived diphenylprolinol had been successfully developed as an effective CSA for the analysis of chiral carboxylic acids.^17^ Therefore, the development of structurally simple efficient receptors for the enantioselective recognition of carboxylic acids is still highly desirable. Taking this fact into account and basing on our experience in the field of the synthesis of a different class of CSAs for the determination of enantiomer ratio, and the application of enantiodiscrimination.^18^ The suitably designed chiral heterocyclic amino alcohol derivatives might be a good candidate because of the existence of multiple H-bonding and the rigid ring of this candidate compounds. Thus, we specifically focus on the further applications of these 2-(diphenylhydroxy) azaheterocycles as chemical probes for the discrimination of carboxylic acids and the measurement of their ee ^1^H NMR spectroscopy.

The synthesis of chiral diphenylmethanol-containing *N*-heterocyclic amino alcohols, in general, was accomplished from commercially available methyl 1-aza-heterocycle-2-carboxylate in a two-step synthesis (the general synthetic procedures are illustrated in ESI Scheme S1†). Four molecular structures of aza-heterocycle-containing diphenylmethanols 1–4 are shown in Fig. 1; in principle, these compounds are suitable candidates for H bonding receptors and they are often used by chemists as enantioselective catalysts in various reactions, and our finding that they are useful CSAs extend their utility.

**Fig. 1:**
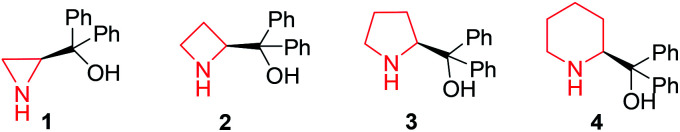
Chiral aza-heterocycle-containing diphenylmethanols 1–4.

We conducted the initial exploratory study using racemic analytes in chloroform-*d* as the most common solvent to determine their abilities to split the signals in the NMR analysis, and explored the binding properties of chiral aza-heterocycle-containing diphenylmethanols 1–4. The (*S*)-pyrrolidinyl diphenylmethanol 3 as CSA has been proved in our previous study,^17^ we had found that the suitable stoichiometries of the host–guest complex, the stoichiometric amounts of racemic mandelic acid and (*S*)-pyrrolidinyl diphenylmethanol 3 molar ratio of 1 : 1 was finally utilized to carry the application for the resolution of discrimination and measurement of carboxylic acids. Thus, equimolar amounts of all of the chiral aza-heterocycle-containing diphenylmethanols, relative to (±)-3,5-difluoro mandelic acid (MA), were found to be sufficient to discriminate between the corresponding enantiomers. The comparing results of these experiments are shown in Table 1, and their ^1^H NMR spectra show that the single peak of α-H has particularly significant splitting. The nonequivalent chemical shift difference (ΔΔ*δ*) of α-H in racemic 3,5-difluoro-MA was calculated to be 0.194 ppm for (*S*)-aziridinyl diphenylmethanol 1, 0.046 ppm for (*S*)-azetidinyl diphenylmethanol 2, 0.050 ppm for (*S*)-pyrrolidinyl diphenylmethanol 3 and 0.033 ppm for (*S*)-piperidinyl diphenylmethanol 4 (Table 1, entries 1–4). Compared with its higher homologues, such as pyrrolidine and piperidine, aziridine and azetidine were seen as more suitable for application as effective NMR discriminating agents for mandelic acids (ESI Fig. S11–S14†). Interestingly, the signal splitting was particularly significant with (*S*)-aziridinyl diphenylmethanol 1, achieving a value of 77.6 Hz (at 400 Hz) for methine proton (C^α^H) signals. The preliminary results evidenced that CSA 1 can act as a more efficient chiral solvating agent towards MA because of its rigid structure of the aziridine ring. Enantiopure aziridinyl diphenylmethanol, *i.e.*, *ent*-1 has also explored its efficacy in binding with racemic 3,5-difluoro-MA, a similar degree of splitting was obtained in the separated peaks (0.184 ppm, Table 1, entry 5).

**Table 1:** Effect of different aza-heterocycle-containing diphenylmethanols as CSAs on the α-proton of racemic 3,5-difluoro-MA

Entry	Aza-heterocycle-containing diphenylmethanols	Probe signal α-H ΔΔ*δ*^a^ (ppm)
1	(*S*)-Aziridinyl diphenylmethanol 1	0.194
2	(*S*)-Azetidinyl diphenylmethanol 2	0.046
3	(*S*)-Pyrrolidinyl diphenylmethanol 3	0.044
4	(*S*)-Piperidinyl diphenylmethanol 4	0.034
5	(*R*)-Aziridinyl diphenylmethanol *ent*-1	0.184

a ΔΔ*δ* = chemical shift non-equivalences for α-H signals.

Having the most active (*S*)-aziridinyl diphenylmethanol 1 in hand, to define the binding interactions, NMR titration experiments were performed. The effect of concentration on the chemical shift differences (ΔΔ*δ*) was examined by the gradual addition of CSA 1 to a solution of (*rac*)-4-MeO-MA (Fig. 2). Baseline resolution was achieved over a considerably wide concentration ratio of [(*S*)-aziridinyl diphenylmethanol 1]/[(*rac*)-4-MeO-MA] (*ca.* 0.25–4.0), with an upfield chemical shift (first upfield and then reach equilibrium). The largest ΔΔ*δ* values of the methane proton were observed when 1 : 1 mixtures of host and guest were used, and it is worth pointing out that the split separation signals of the methoxy group of (*rac*)-4-MeO-MA are also observed (see ESI Fig. S16†).

**Fig. 2:**
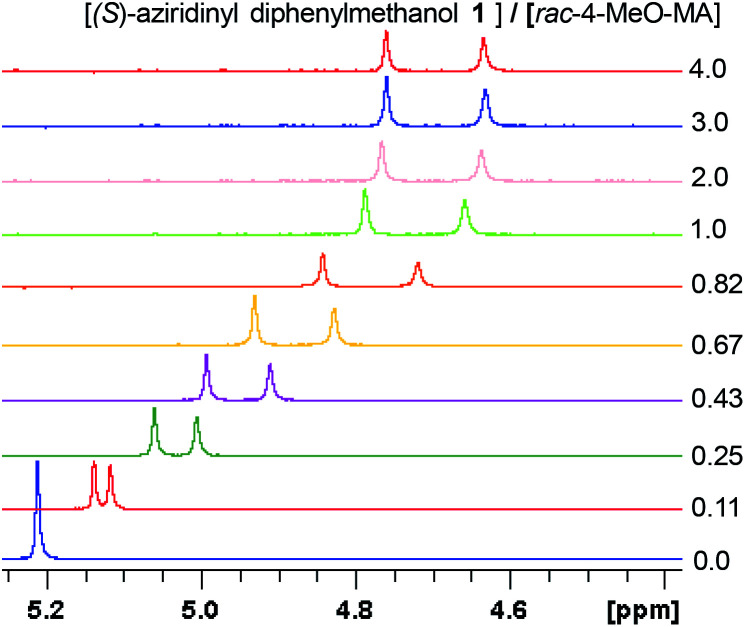
Part of the ^1^H NMR spectra of (*rac*)-4-MeO-MA in the presence of different molar amounts of (*S*)-aziridinyl diphenylmethanol 1.

The stoichiometries of the CSA and guests were further established by the ^1^H NMR analysis using Job plots. Fig. 3 plots the chemical shift non-equivalencies (Δ*δ*) of 4-MeO-MA enantiomers observed for the CSA 1 as a function of molar ratio. In most cases, the enantiomeric separation increases sharply as the molar ratio increases from 0 to about 0.5 and then steadily declines. The measured Δ*δ* values represent the shift in the NMR signal of the α-H proton of (*R*)-4-MeO-MA in the presence of CSA. The *X* represents the molar fraction of the guest in the mixture. The Job plots of *X* × Δ*δ versus* the molar fraction (*X*) of (*R*)- or (*S*)-4-MeO-MA in the mixture were obtained at 10 mM in CDCl_3_, which showed a maximum at *X* = 0.5, and the best baseline resolution was both achieved with a 1 : 1 ratio of CSA 1/4-MeO-MA, as shown in Fig. 3. This further indicated that CSA 1 and the acid bind in a 1 : 1 complex under these conditions.

**Fig. 3:**
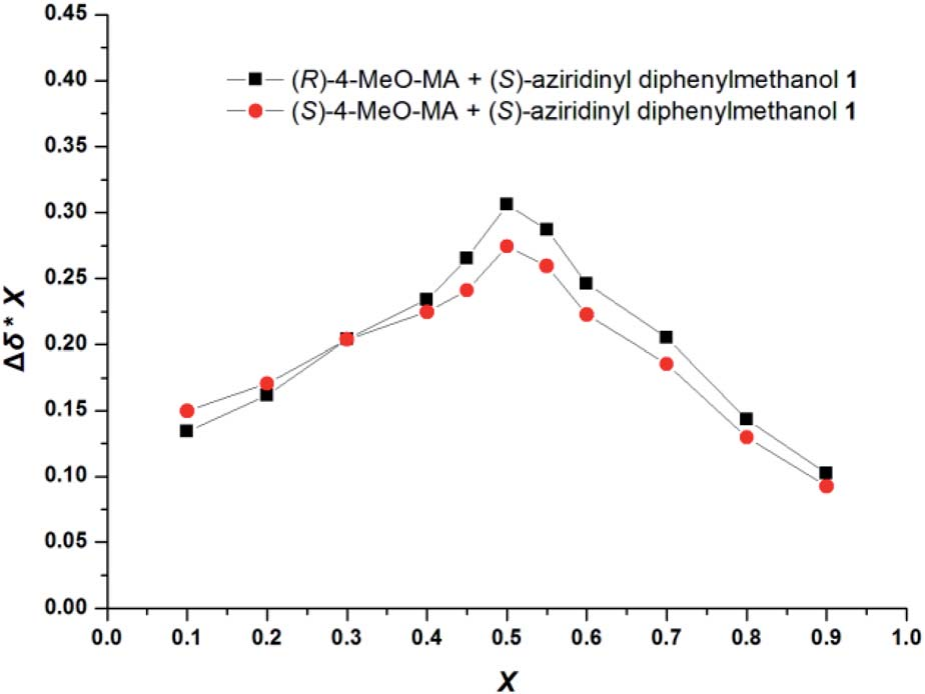
Job plots of (*S*)-aziridinyl diphenylmethanol 1 with (*R*)-4-MeO-MA and (*S*)-4-MeO-MA. Δ*δ* stands for the chemical shift change of the α-H proton of (*R*)- and (*S*)-4-MeO-MA in the presence of (*S*)-aziridinyl diphenylmethanol 1. *X* stands for the molar fraction of the (*S*)-aziridinyl diphenylmethanol 1 (*X* = [(*S*)-aziridinyl diphenylmethanol 1]/[(*S*)-aziridinyl diphenylmethanol 1] + [4-MeO-MA]). The total concentration is 10 mM in CDCl_3_.

After obtaining the most active (*S*)-aziridinyl diphenylmethanol 1 and using the optimal conditions, in the next step of studies, the influence of various electron-donating and electron-accepting substituents in the racemic alpha-substituted carboxylic acids on the chiral recognition was examined and evaluated. All the experiments were carried out by adding a certain amount of solution of racemic carboxylic acid in CDCl_3_ (10 mM) to a solution of (*S*)-aziridinyl diphenylmethanol 1 in CDCl_3_ (10 mM). Immediately after each addition, the ^1^H NMR spectrum was acquired in a 400 MHz spectrometer at room temperature. As shown in Table 2, all racemic analytes can be well-resolved (ΔΔ*δ* values up to 0.194 ppm, 77.6 Hz). To our delight, the ΔΔ*δ* values of the α-H signals were large enough to give very good baseline resolution for most tested aromatic carboxylic acids containing α-OH (Table 2, entries 1–18). Notably, fluorine-substituted aromatic carboxylic acids (Table 2, entries 4–7, 9 and 11) almost showed more baseline resolution and had larger ΔΔ*δ* values than other diverse substituents such as chlorine and bromine, irrespective of their electronic nature or positions, the role of fluorine in the aromatic MA systems for the strongly enhance the binding ability of the aziridinyl diphenylmethanol (*S*)-CSA 1, which results in a better splitting of the NMR signal of the corresponding guests.

**Table 2:** ^1^H NMR chemical shift non-equivalent values (ΔΔ*δ* in ppm and Hz, 400 MHz) of different racemic carboxylic acids in presence of equimolar amounts of (*S*)-aziridinyl diphenylcarbinol 1 at 25 °C in CDCl_3_^a^

Entry	Carboxylic acids	ΔΔ*δ*^b^	Spectra
1	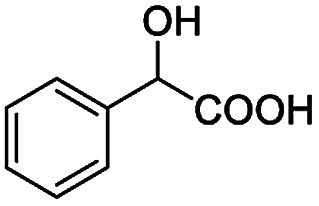	0.134, 53.6	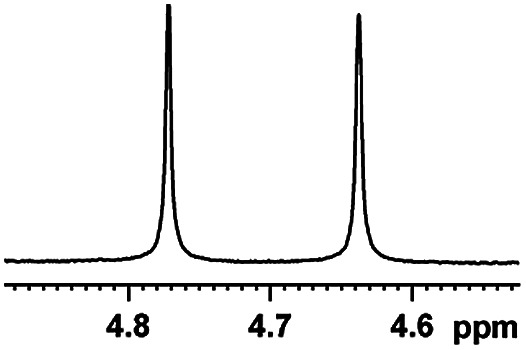
2	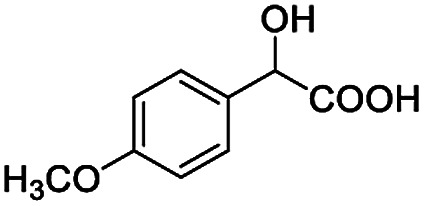	0.137, 54.8	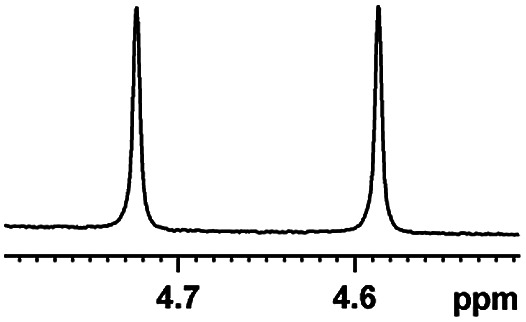
3	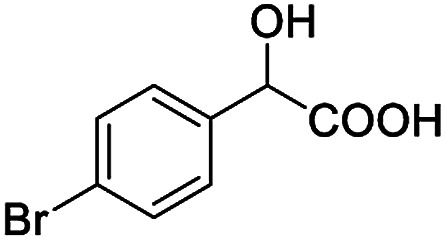	0.100, 40.0	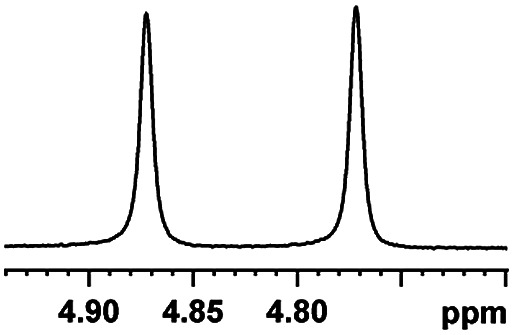
4	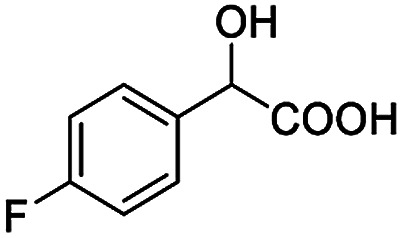	0.148, 59.2	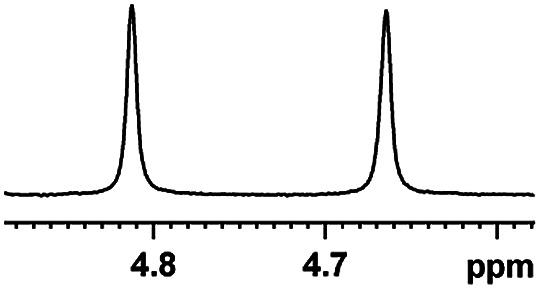
5	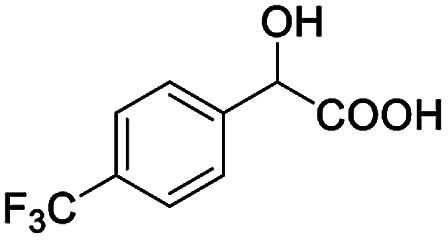	0.138, 55.2	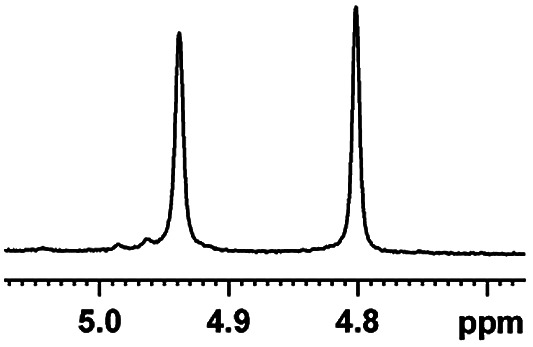
6	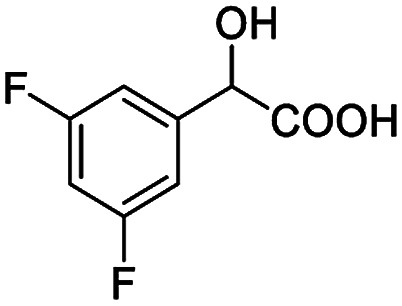	0.194, 77.6	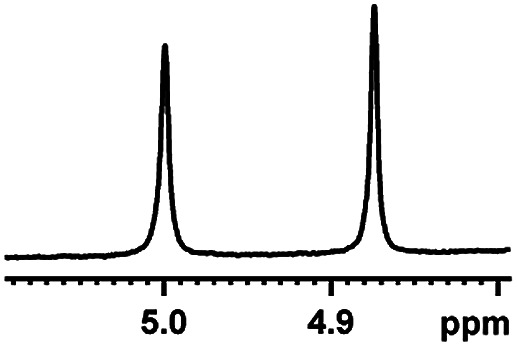
7	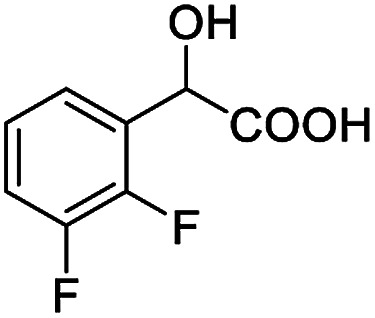	0.125, 50.0	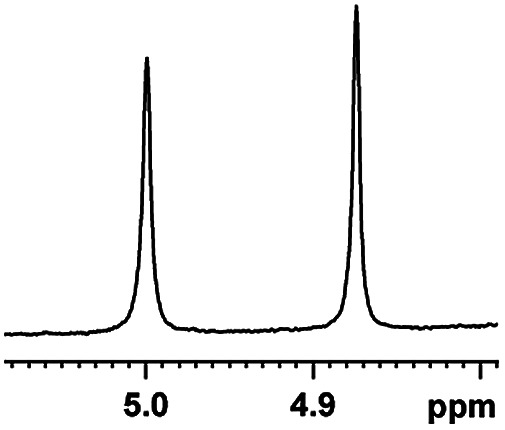
8	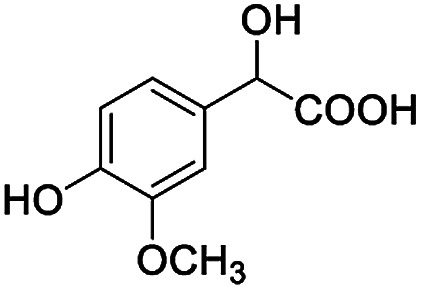	0.121, 48.4	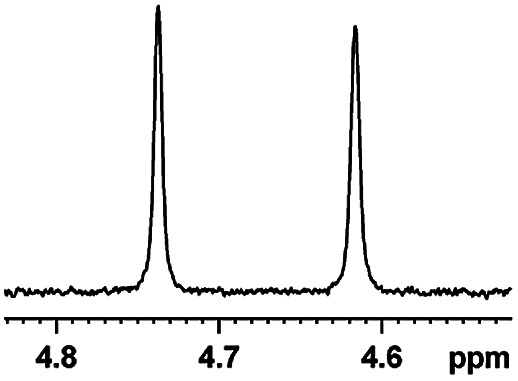
9	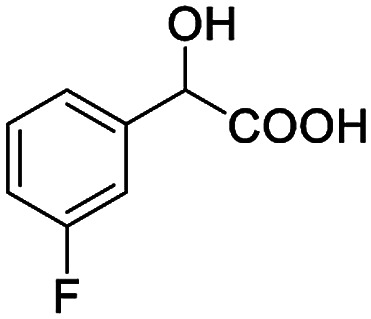	0.134, 53.6	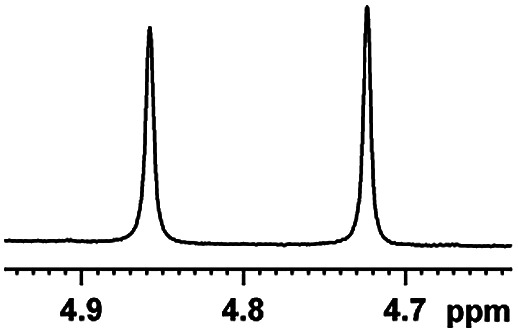
10	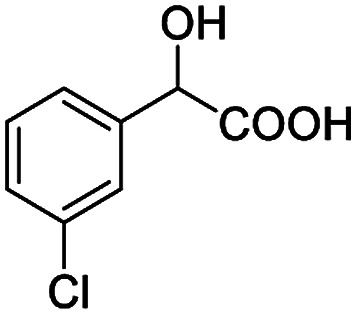	0.118, 47.2	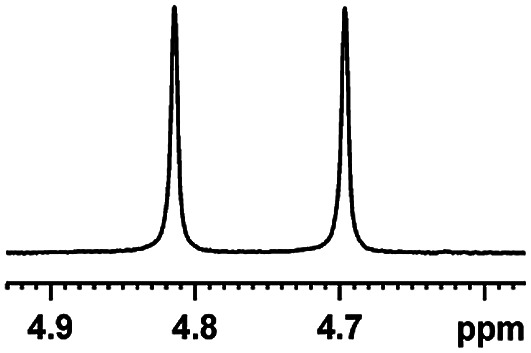
11	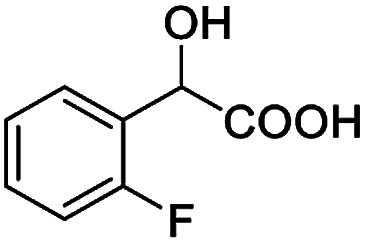	0.109, 43.6	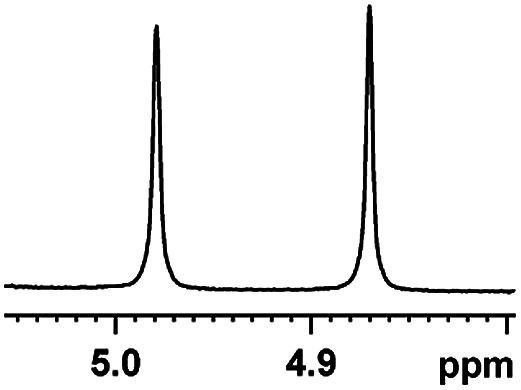
12	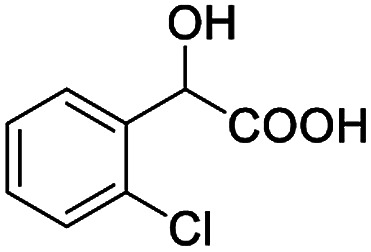	0.050, 20.0	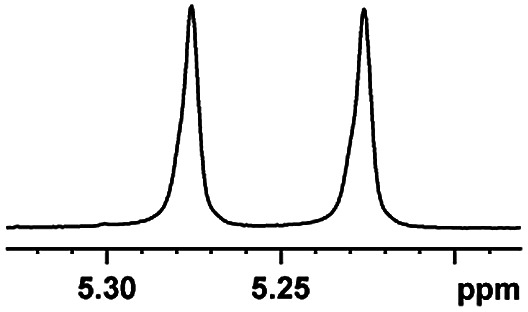
13	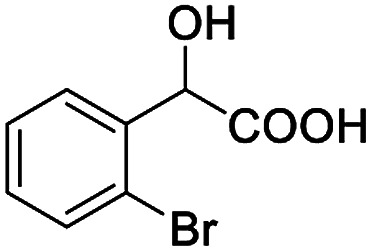	0.053, 21.2	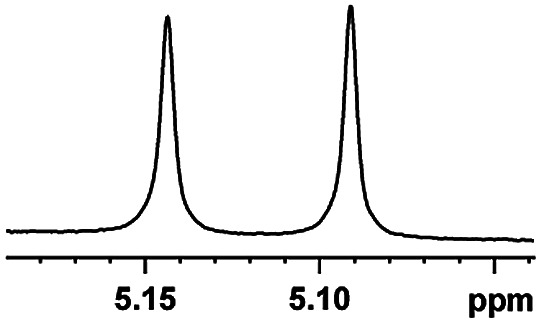
14	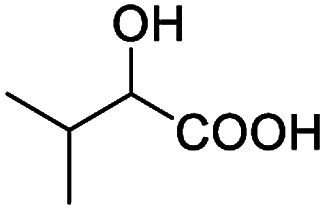	0.111, 44.4	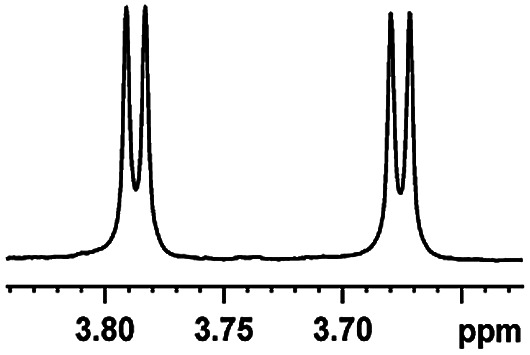
15	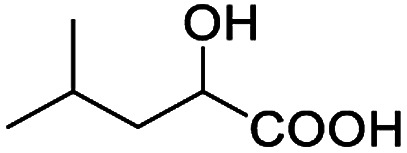	0.084, 33.6	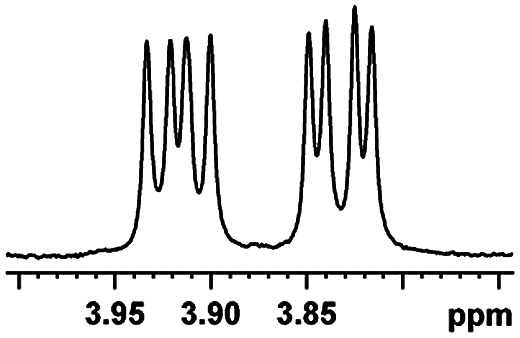
16	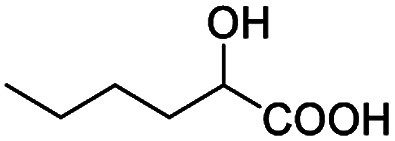	0.068, 27.2	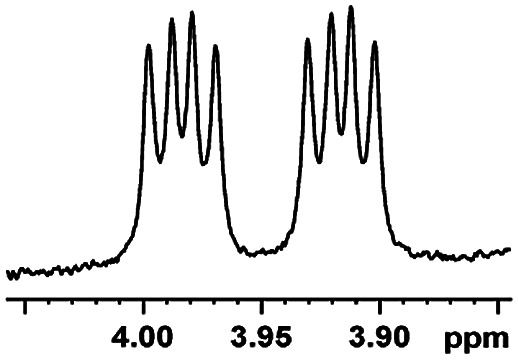
17	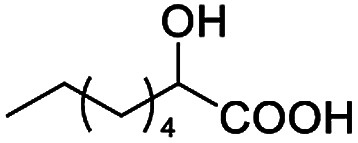	0.058, 23.2	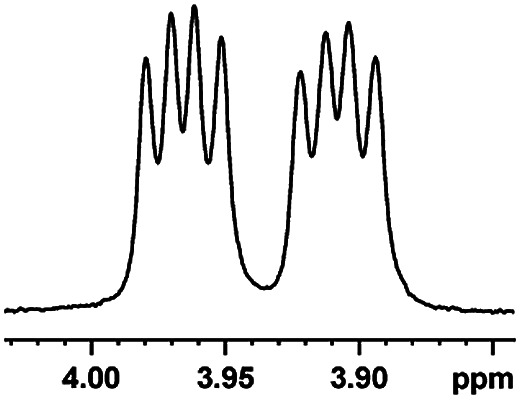
18	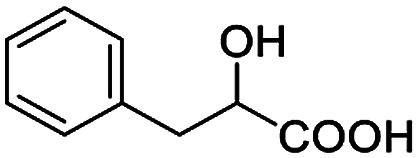	0.079, 31.6	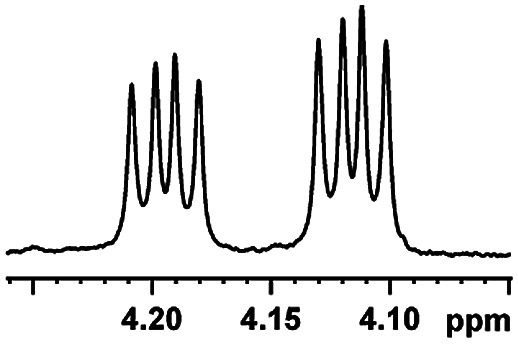
19	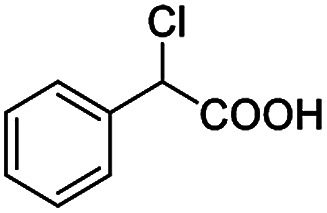	0.150, 60.0	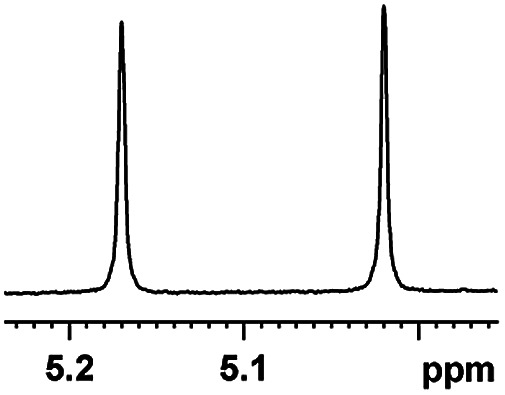
20	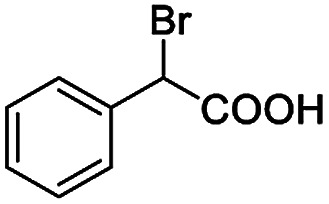	0.101, 40.4	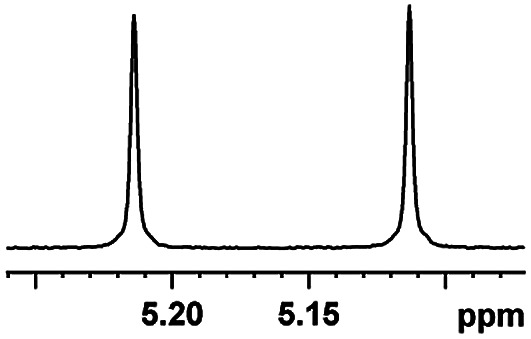
21	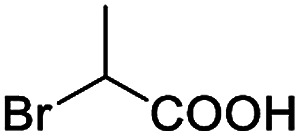	0.012, 4.8	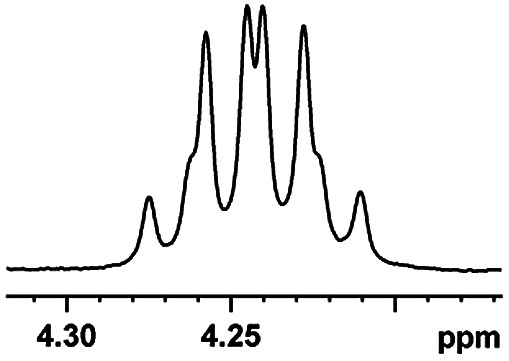
22	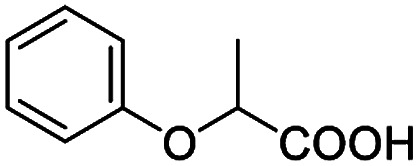	0.104, 41.6	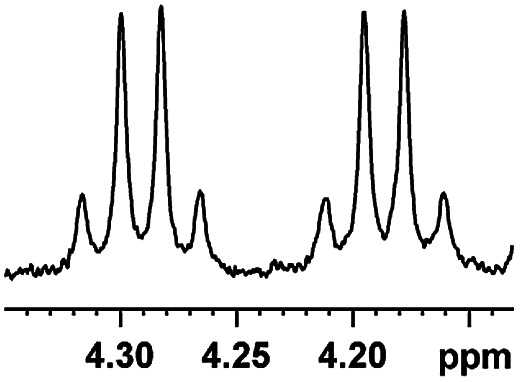
		0.188, 75.2^d^	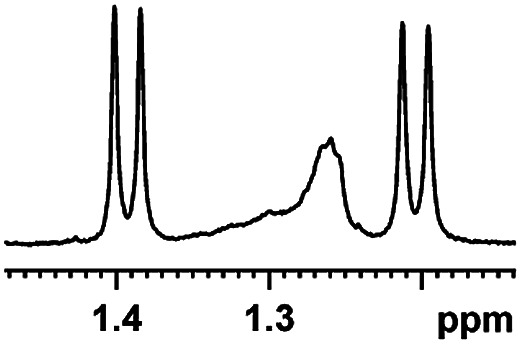
23	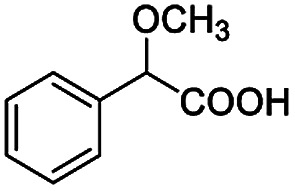	0.039, 15.6	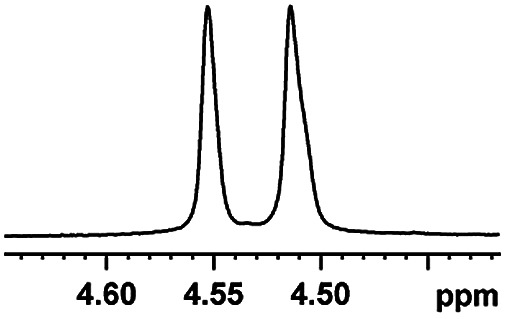
		0.052, 20.8^c^	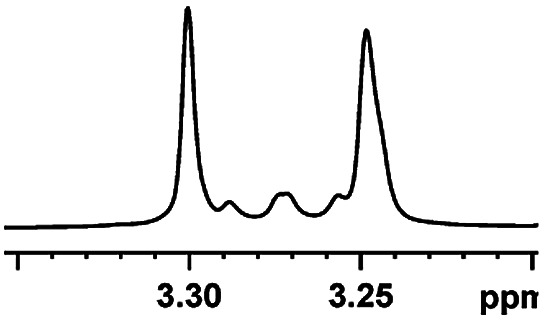
24	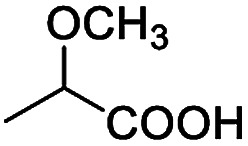	0.041, 16.4^c^	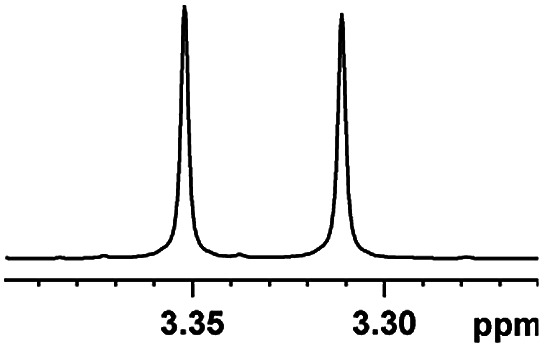
		0.049, 19.6^d^	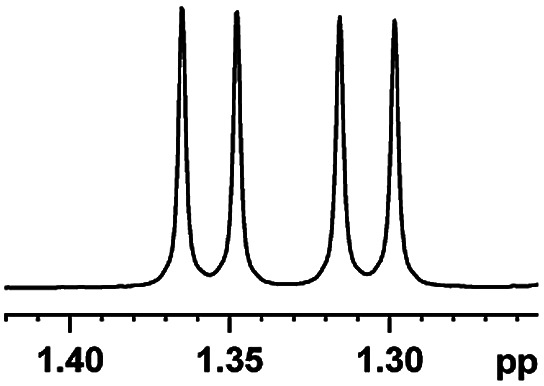
25	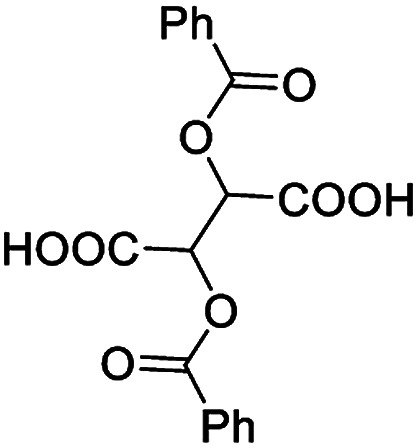	0.029, 11.6^e^	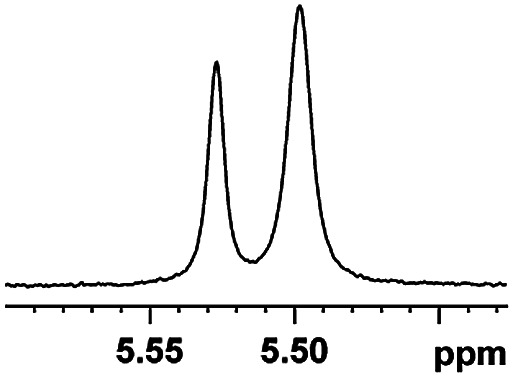

a All samples were prepared by mixing 1 : 1 of carboxylic acids (10 mM in CDCl_3_) with (*S*)-CSA 1 in NMR tubes, and the spectra is recorded at 25 °C on a 400 MHz spectrometer.

b ΔΔ*δ* values in ppm and Hz for α-H are shown.

c Chemical shift non-equivalences of the α-methoxy group.

d Chemical shift non-equivalences of the α-methyl group.

e Use of 2 equiv. of (*S*)-CSA 1 for the dicarboxylic acid.

In addition, we further evaluated the chiral discriminating abilities of (*S*)-aziridinyl diphenylcarbinol 1 for aliphatic 2-hydroxymonocarboxylic acids as guests, and these 2-hydroxycarboxylic acids are known for their relationship with particular human illnesses, especially but not only with inherited metabolic diseases.^19^ 2-hydroxy-3-methylbutyric acid (2-H-3-MBA), 2-hydroxyisocaproic acid (2-HICA), and 2-hydroxyoctanoic acid (2-HOA) are related to certain types of organic acidurias. For example, an increased amount of 2-HICA has been found in patients with the Zellweger syndrome, a congenital disorder.^20^ The inspection of results presented in the fragments of ^1^H NMR spectra (0.111 ppm, 44.4 Hz; 0.084 ppm, 33.6 Hz; 0.058 ppm, 23.2 Hz; 0.058 ppm, 23.2 Hz, and 0.079 ppm, 31.6 Hz, Table 2, entries 14–18) indicates that the robust (*S*)-aziridinyl diphenylmethanol 1 as CSA can give a good baseline resolution.

Moreover, clearly resolved signals from α-H of 2-halogenocarboxylic acids are observed with almost baseline separation, and their partial spectra are shown in Table 2 (entries 19–20 (0.150 ppm, 60.0 Hz and 0.101 ppm, 40.4 Hz)). Interestingly, similar fluorine substituted effect on the ability of discrimination was observed in the aforementioned racemic mandelic derivatives. 2-Bromo propionic acid displayed poor enantiodiscriminating ability (0.012 ppm, 4.8 Hz, Table 2, entry 21).

Besides, when the free α-hydroxyl group was replaced by the methoxy group, the chiral discrimination was also observed for OMe and methine proton (C^α^H) signals (Table 2, entries 22–24), while the dicarboxylic acid derivative was tested, 2 equiv. of (*S*)-CSA 1 was used and the ΔΔ*δ* value decreased slightly (Table 2, entry 25).

The fluorine element has polar and steric properties as a substituent, and the effects that fluorinated groups can have on the physical and chemical properties of molecules have increased the number of new methods for incorporating fluorine into target compounds. Since the ^19^F NMR frequency and sensitivity are similar to the proton, it is convenient to transition from the proton to fluorine NMR experiments. Then, we recorded the ^19^F–{^1^H} NMR spectra of fluorinated carboxylic acids. The results are listed in Table 3 (entries 1–8). It can be seen that the fluorine signal separation is visible for fluorinated MAs (entries 1–6). As for fluorinated substrates, obtaining a distinguishable NMR split signal highly depends on the distance; therefore, 4-CF_3_-substituted MA showed the lowest fluorine NMR chemical shift with 0.015 ppm, while the α-fluorine phenylacetic acid gave the well-split F signal with 0.859 ppm chemical shift difference value (entry 7 *vs.* 8).

**Table 3:** ^19^F NMR chemical shift non-equivalent values (ΔΔ*δ* in ppm and Hz, 376 MHz) of fluorine-containing α-substituted carboxylic acids in the presence of equimolar amounts of (*S*)-aziridinyl diphenylcarbinol 1 at 25 °C in CDCl_3_

Entry	Carboxylic acids	ΔΔ*δ*	Spectra
1	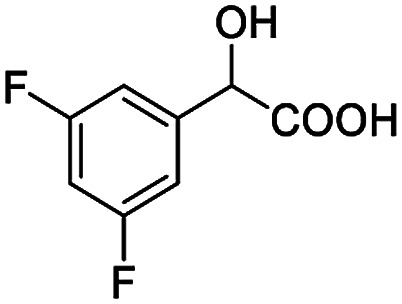	0.076, 28.6	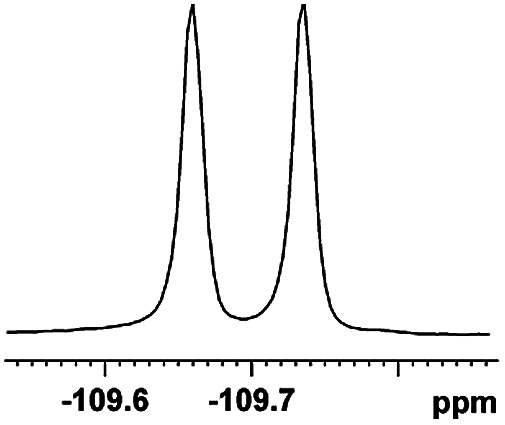
2	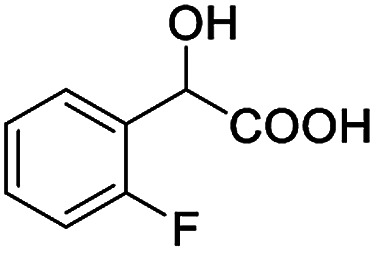	0.057, 21.5	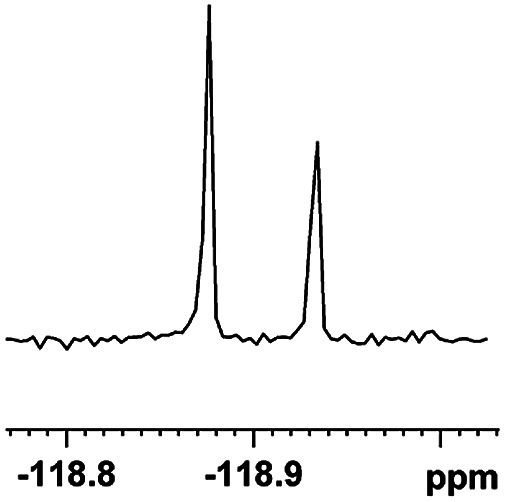
3	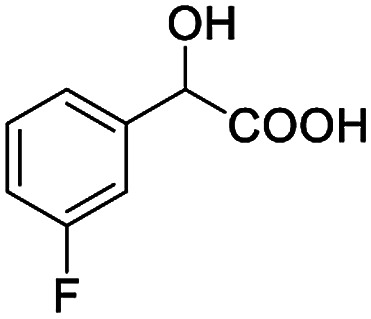	0.054, 20.0	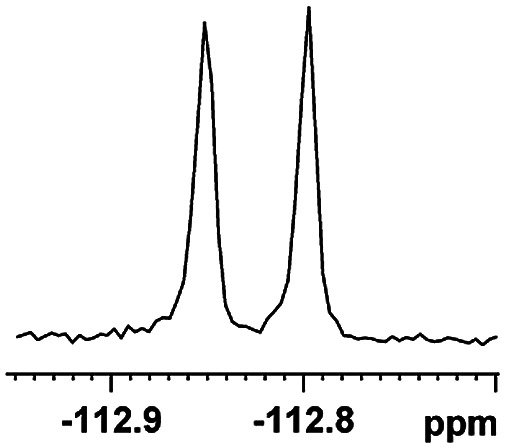
4	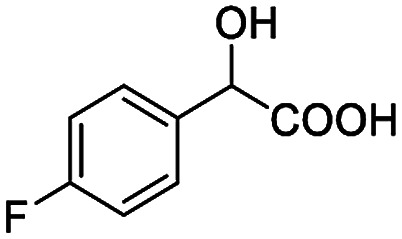	0.256, 96.2	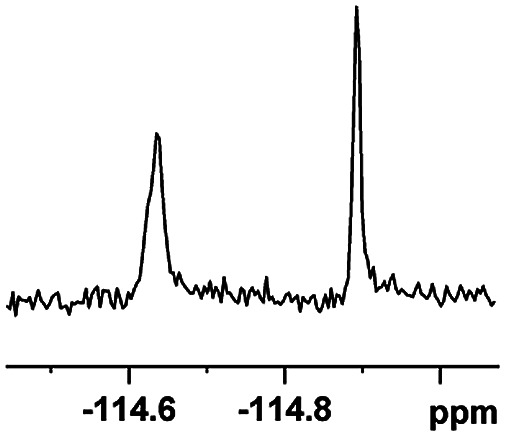
5	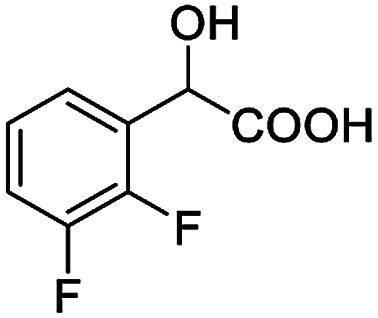	0.056, 21.3^a^	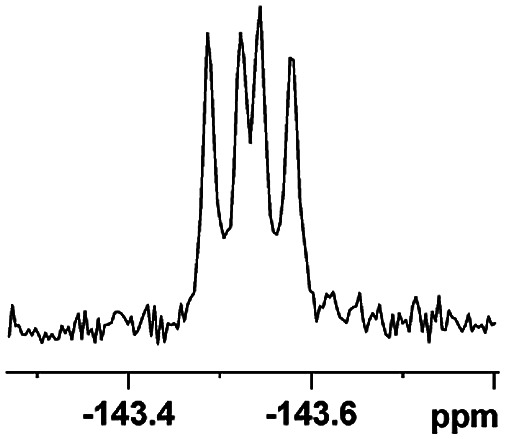
		0.054, 20.4^b^	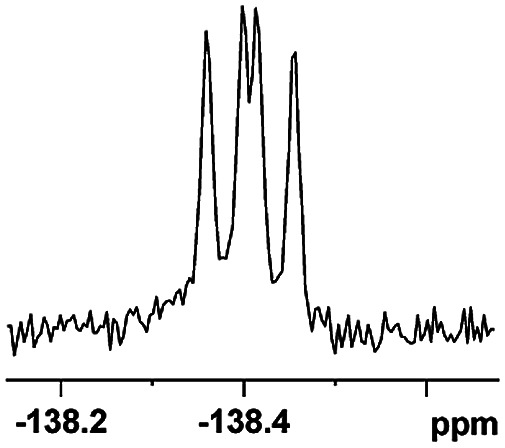
6	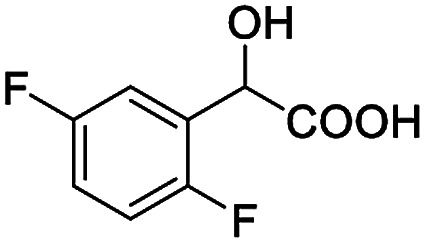	0.068, 25.9^a^	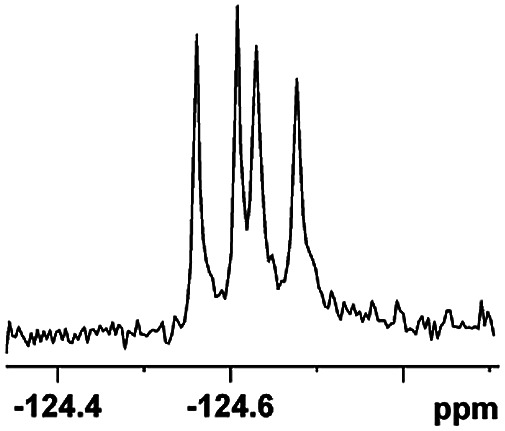
		0.080, 30.2^b^	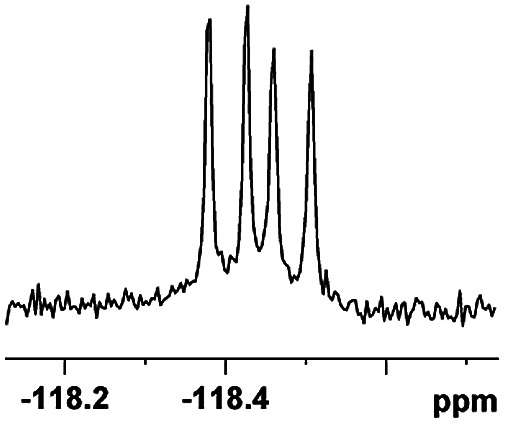
7	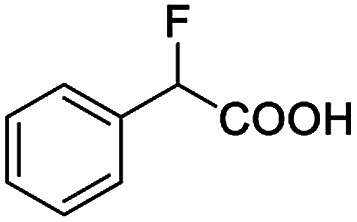	0.859, 323.3	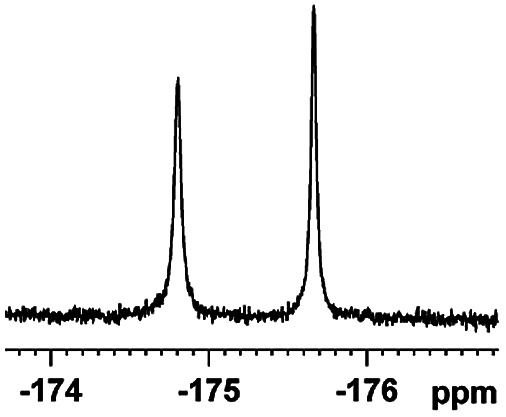
8	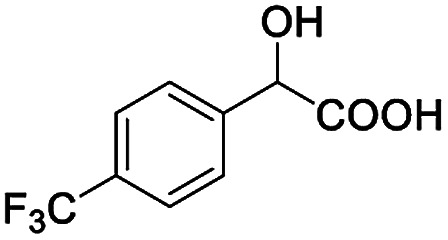	0.015, 5.6	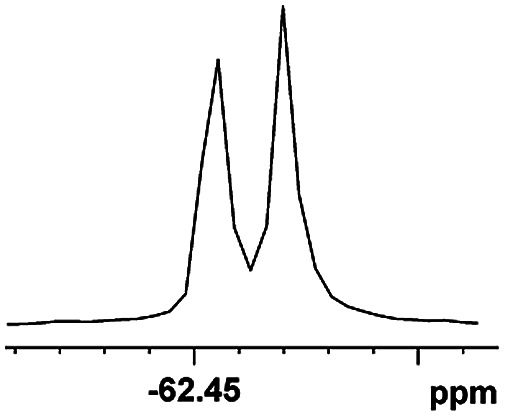

a The proton-decoupled ^19^F NMR spectra was obtained from *o*-substituted fluorine.

b The proton-decoupled ^19^F NMR spectra was obtained from *m*-substituted fluorine.

Now that (*S*)-aziridinyl diphenylcarbinol 1 had been established to demonstrate efficiently chiral discriminating abilities towards α-hydroxyl acids *via*^1^H NMR spectroscopy, to further demonstrate the practical utility of (*S*)-aziridinyl diphenylcarbinol 1 as a CSA for enantiomeric determination. It allowed determining the enantiomeric composition of multiple nonracemic 4-MeO-MA samples by the simple integration of the split signals, and samples containing (*R*)-4-MeO-MA with 80, 60, 40, 20, 0, −20, −40, −60, −80 and −100% were prepared in the presence of (*S*)-aziridinyl diphenylcarbinol 1, and their ^1^H NMR spectra were measured. Enantiomeric excesses of all samples were calculated based on the integration of the ^1^H NMR signals of the α-H signal protons of the enantiomers of (*R*)- and (*S*)-4-MeO-MA. The overlaid partial ^1^H NMR spectra and linear correlation between the theoretical (*X*) and observed ee% (*Y*) are shown in Fig. 4. The linear relationship between the NMR-determined values and those gravimetrically determined values is excellent with *R*^2^ = 0.99995. The average absolute error in the ee measurement plotted in Fig. 4 is within 1%.

**Fig. 4:**
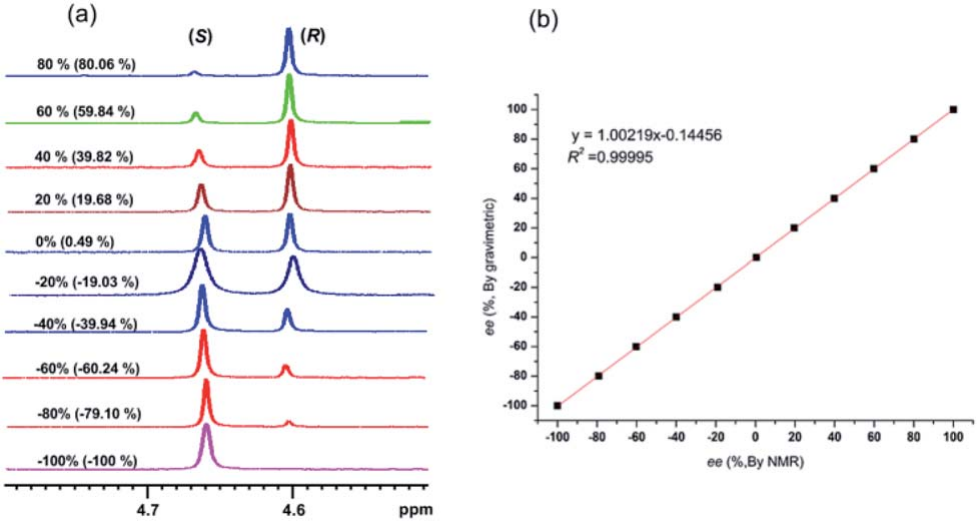
(a) Selected regions of the ^1^H NMR spectra of different optical purities 4-MeO-MA samples (ee% = *R*% − *S*%) with (*S*)-aziridinyl diphenylcarbinol 1 in CDCl_3_; (b) linear correlation between ee values determined by gravimetry (*Y*) and values were defined in terms of (*R*)-4-MeO-MA (*X*), *R*^2^ = correlation coefficient.

## Conclusions

In summary, we have demonstrated a general and efficient chiral solvation of carboxylic acids with a chiral aza-semi crown amino alcohol, (*S*)-aziridinyl diphenylcarbinol 1. Efficient peak separation of racemic analytes was achieved after the addition of small amounts of the CSA in CDCl_3_ at room temperature (25 examples), the fluorinated carboxylic acids were further investigated, with a good level of discrimination by the ^19^F NMR spectroscopic analysis. Moreover, quantitative analysis was performed according to the integration of the ^1^H NMR spectra, and an excellent linear relationship was observed between the experimental and observed values of ee. The easy accessibility of the CSA (1–4) from commercially available methyl 1-aza-heterocycle-2-carboxylate should make this methodology very attractive for practical applications as CSAs for alpha-substituted carboxylic acids. The mode of recognition was based on effective hydrogen bond formation. It has shown the possibility that this class of CSAs to be used for determining the optical purity of unknown samples as well as other supramolecular and macrocyclic host-based applications.

## Conflicts of interest

There are no conflicts to declare.

## Supplementary Material

RA-010-D0RA06312F-s001
