# Supplementary material for: Azaheterocyclic diphenylmethanol chiral solvating agents for the NMR chiral discrimination of alpha-substituted carboxylic acids
Source: RSC Adv. 2020 Sep 18;10(57):34605–11. doi: 10.1039/d0ra06312f (PMC9056771; doi:10.1039/d0ra06312f)
Supplement: RA-010-D0RA06312F-s001 [file RA-010-D0RA06312F-s001.pdf]

Electronic Supplementary Material (ESI) for RSC Advances.
This journal is © The Royal Society of Chemistry 2020

Supporting Information

Azaheterocyclic diphenylmethanol chiral solvating agents for the NMR chiral discrimination of alpha-substituted carboxylic acids

Gao-Wei Li,[a,b] Xiao-Juan Wang, *[a] Dan-Dan Cui, [a] Yu-Fei Zhang, [a] Rong-Yao Xu, [a] Shuai-Hua Shi, [a] Lan-Tao Liu, *[a] Min-Can Wang,[b] Hong-Min Liu , [b] and Xin-Xiang Lei *[c]

[a] *College of Chemistry and Chemical Engineering and Henan Engineering Laboratory of Green Synthesis for Pharmaceuticals, Shangqiu Normal University, Shangqiu, 476000, P.R. China.*
[b] *School of Pharmaceutical Sciences, Zhengzhou University, Zhengzhou, 450001, P.R. China.*
[c] *School of Pharmaceutical Sciences, South-Central University for Nationalities, Wuhan, 430074, P.R. China.*

1. General methods 2
2. General procedure for the synthesis of the chiral solvating agents 2
2.1. Synthetic procedures of compound *N*-protected aza-heterocyclic diphenylmethanols 2
2.2. General procedure for the deprotection by hydrolysis reaction 3
3. Determination of enantiomeric purity of mandelic acid 4
4. Discrimination ability of CSA 1 toward racemic guests 1-25 4
5. ^{1}H NMR, ^{13}C NMR spectra of CSAs 5
6. ^{1}H NMR spectroscopy CSA 1-4 and racemic 3,5-difluoro-mandelic acid 10
7. ^{1}H NMR spectroscopy (*S*)-aziridinyl diphenylmethanol and various racemic α-substituted carboxylic acids 13
8. ^{19}F NMR spectroscopy (*S*)-aziridinyl diphenylmethanol and fluorine-containing α-substituted carboxylic acids 26

1. General methods

Solvents were dried with standard methods and freshly distilled prior to use if needed. Optical rotations were measured with Perkin Elmer, model 341 Polarimeter at 20 °C in $CHCl_3$. CSA **3** was prepared from commercial methyl 1-tritylaziridine-2-carboxylate, others chemicals were either purchased or purified by standard techniques. Melting pointswere obtained with a Yuhua X-5 micromelting point apparatus and uncorrected. ^1H NMR, ^{13}C NMR and ^{19}F NMR spectra were measured on 400 MHz Brucker spectrometer in $CDCl_3$ solutions with tetramethylsilane (TMS). *J* values are given in Hz. All spectra were recorded using 16 scans at 298 K. An exponential window function with a line-broadening factor of 1 Hz was applied to the FID before Fourier transformation. Column chromatography was performed using Silica gel (300-400 mesh).

2. General procedure for the synthesis of the chiral solvating agents

The chiral aza-heterocycle-containings diphenylmethanols can be readily carried out in a two-step sequence in good yield from commercially available methyl 1-aza-heterocycle-2-carboxylate with Grignard reagent and hydrolysis reaction, the route of synthesis as shown in Scheme S1.

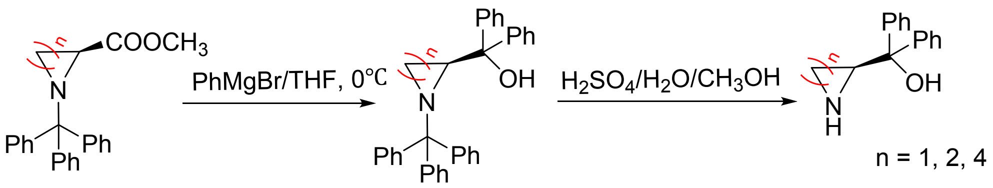

Scheme S1. Preparation and structures of aza-heterocycle-containing diphenylmethanols.

2.1. Synthetic procedures of compound *N*-protected aza-heterocyclic diphenylmethanols

To a Grignard reagent solution prepared from 6.3 mL (60 mmol) of bromobenzene in 5 mL of THF and 1.46 g (60 mmol) of magnesium in 10 mL of THF was gradually added 15 mmol of methyl 1-aza-heterocycle- 2-carboxylate dissolved in 5 mL of THF at 20 °C. The mixture was then allowed to reach room temperature. After stirring for 12 h, the reaction was quenched with saturated aqueous NH_4Cl (8 mL) at 0 °C. The product was separated and the aqueous phase extracted with ethyl acetate (3×10 mL). The combined organic phases were washed with brine (10 mL), dried over Na_2SO_4, and concentrated under reduced pressure. The resulting residue was purified by column chromatography with petroleum ether /ethyl acetate as the developing solvent to give the *N*-protected aza-heterocyclic diphenylmethanols.

2.2. General procedure for the deprotection by hydrolysis reaction

N-protected aza-heterocyclic diphenylmethanols (3 mmol) was dissolved in $H_2SO_4/H_2O/CH_3OH$ (3/8/60, 18 mL). The solution was stirred at room temperature for 24 h, the white precipitate formed was removed by filtration from the mixture, and then to the filtrate NaOH 30% w/w solution was carefully added simultaneously to adjust the solution mixture to around 10 pH and the solution was extracted with ethyl acetate (3 × 10 mL), the combined extracts were dried over Na_2SO_4. The organic phase was then concentrated in vacuo and the residue was purified by silica gel column chromatography with petroleum ether /ethyl acetate (4:1, v/v) as eluent to afford (*S*)-CSA-**1** as a white solid.

(*S*)-aziridinyl diphenylmethanol **1**: white solid, m. p. =162-163 °C; $[\alpha]^{25}_{D}$ = -20.5 (*c* 0.294, $CHCl_3$); ^{1}H NMR (400 MHz, $CDCl_3$) δ 7.46 – 7.23 (m, 10H), 2.92 (s, 1H), 1.87 (d, *J* = 5.6 Hz, 1H), 1.75 (d, *J* = 3.5 Hz, 1H); ^{13}C NMR (100 MHz, $CDCl_3$) δ 147.3, 145.2, 128.2, 128.1, 127.1, 127.1, 126.5, 126.3, 74.3, 37.0, 22.0; HRMS (EI-TOF): m/z Calculated for $C_{15}H_{15}NO$ (M^+): 225.1154; Found: 225.1167.

(*R*)-aziridinyl diphenylmethanol ***ent*-1**: white solid, m. p. =160-162 °C; $[\alpha]^{25}_{D}$ = +23.4 (*c* 0.20, $CHCl_3$); ^{1}H NMR (400 MHz, $CDCl_3$) δ 7.44 – 7.23 (m, 10H), 2.92 (s, 1H), 1.88 (d, *J* = 5.6 Hz, 1H), 1.74 (d, *J* = 3.6 Hz, 1H); ^{13}C NMR (100 MHz, $CDCl_3$) δ 147.3, 145.1, 128.2, 128.1, 127.18, 127.17, 126.5, 126.3, 74.3, 37.0, 22.1.

(*S*)-azetidinyl diphenylmethanol **2:** white solid, m. p. =112-113 °C; $[\alpha]^{25}_{D}$ = -73.4 (*c* 0.30, $CHCl_3$); ^{1}H NMR (400 MHz, $CDCl_3$): δ 7.43 − 7.16 (m, 10H), 4.91 (t, *J* = 8.0 Hz, 1H), 3.61 (q, *J* = 7.2 Hz, 1H), 3.18 (ddd, *J* = 8.4, 7.2, 3.2 Hz, 1H), 2.42 − 2.33 (m, 1H), 198 − 1.90 (m, 1H); ^{13}C NMR (100 MHz,$CDCl_3$): δ 146.3, 143.3, 128.1, 128.0, 126.7, 126.6, 126.3, 125.9, 76.5, 64.7, 42.3, 21.9; HRMS (EI-TOF): m/z Calculated for $C_{16}H_{17}NO$ (M^+): 239.1310; Found:239.1322.

(*S*)-pyrrolidinyl diphenylmethanol **3:** The compound was purchased from J&K without purification.

(*S*)-piperidinyl diphenylmethanol **4**: white solid, m. p. =92-95 °C; $[\alpha]^{25}_{D}$ = -80.5 (*c* 0.250, $CHCl_3$); ^{1}H NMR (400 MHz, $CDCl_3$) δ 7.62 (dd, *J* = 8.8, 1.6 Hz, 2H), 7.46 (dd, *J* = 8.8, 1.6 Hz, 2H), 7.39 – 7.10 (m, 6H), 4.31 (br, 1H), 3.53 (dd, *J* = 10.8, 2.8 Hz, 1H), 3.01 (dq, *J* = 10.8, 2.0 Hz, 1H), 2.72 (dt, *J* = 11.7, 2.7 Hz, 1H), 1.76 – 1.54 (m, 3H), 1.45 – 1.20 (m, 4H); ^{13}C NMR (100 MHz, $CDCl_3$) δ 146.3, 144.2, 128.5, 127.9, 126.8, 126.3, 126.0, 125.5, 78.5, 61.7, 46.7, 25.6, 25.5, 24.5;

HRMS (EI-TOF): m/z Calculated for $C_{18}H_{21}NO$ (M^+): 267.1623; Found:267.1589.

3. Determination of enantiomeric purity of mandelic acid

To determine the enantiomeric purity of the carboxylic acids, ten 4-MeO-MA samples with-100%, -80%, -60%, -40%, -20%, 0%, 20%, 40%, 60%, 80%*ee* were prepared at a concentration of 10 mM in $CDCl_3$, respectively, expressed as % *R* in the data. The CAS **1** was also dissolved in $CDCl_3$ at a concentration of 10 mM. Then 250 µL of CAS **1** and 250 µL of 4-MeO-MA with different *ee*'s were mixed in the NMR tube generating a total concentration of 10 mM with a molar ratio of 1:1. Then the enantiomeric purity of the carboxylic acids was determined by ^1H NMR method. The plotting of gravimetric *ee* value (y axis) versus NMR observed *ee* value (x axis) presented excellent linearity with R^2 =0.99995.

4. Discrimination ability of CSA 1 toward racemic guests 1-25

At first, CSA **1**, and the guests were separately dissolved in $CDCl_3$ with a concentration of 10 mM. Then, 0.25 mL of CSA **1** and 0.25 mL guest were added to NMR tubes, so that the total volume was 0.5 mL, and the concentration of CSA **1** and guest was 10 mM. The ^1H NMR spectra of all samples were recorded on a 400 MHz spectrometer.

5. ^{1}H NMR, ^{13}C NMR spectra of CSAs

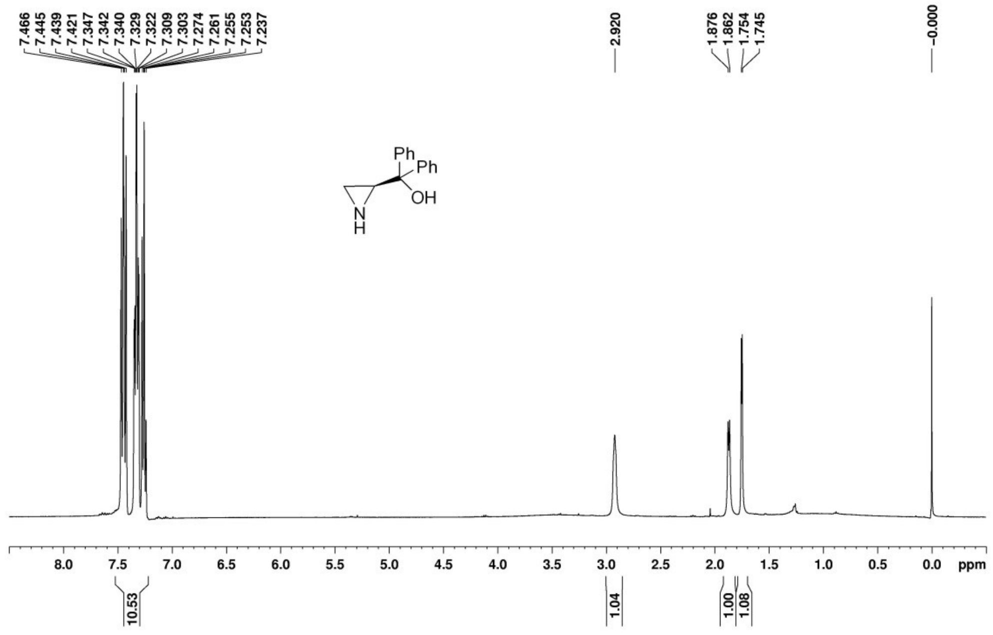

Figure S1. ^{1}H NMR (400 MHz, $CDCl_3$) of (*S*)-aziridinyl diphenylmethanol 1.

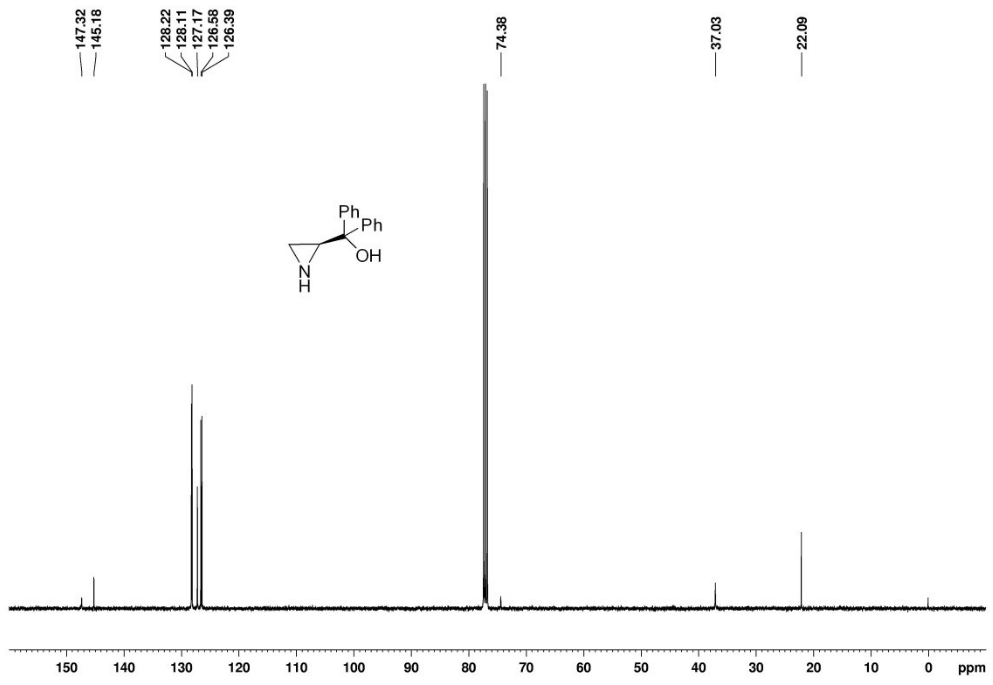

Figure S2. ^{13}C NMR (100 MHz, $CDCl_3$) of (*S*)-aziridinyl diphenylmethanol 1.

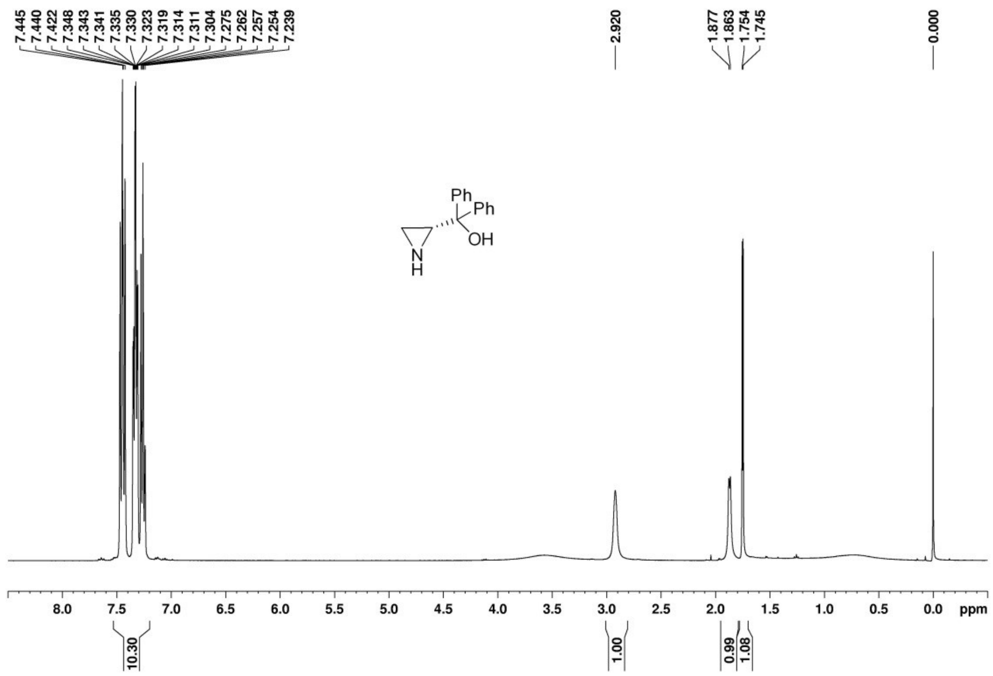

Figure S3. **^{1}H NMR (400 MHz, $CDCl_3$) of (*R*)-aziridinyl diphenylmethanol *ent*-1.**

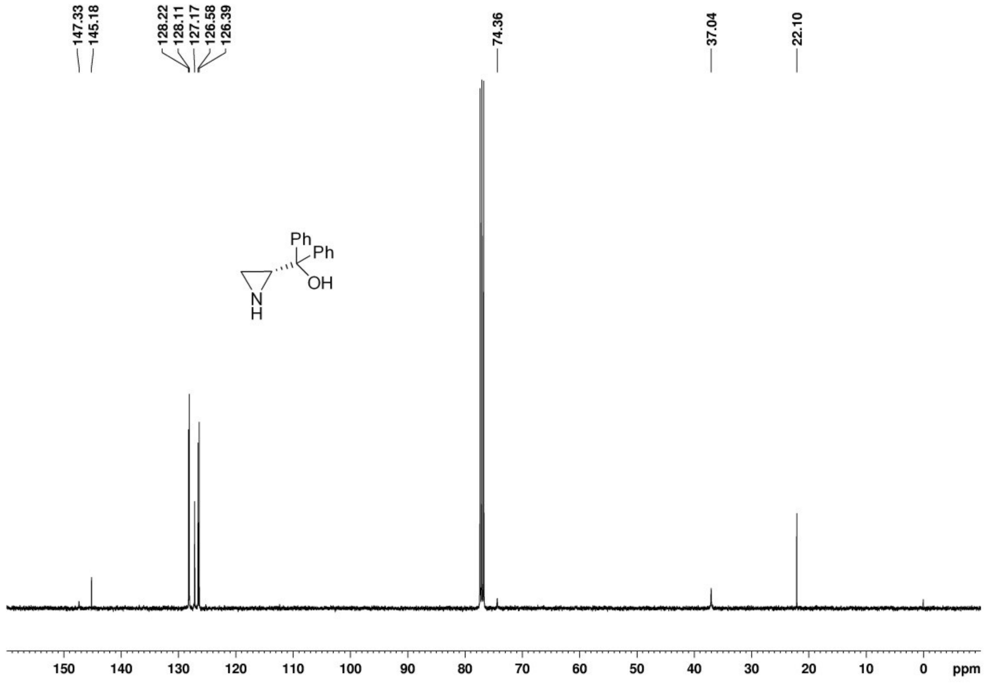

Figure S4. **^{13}C NMR (100 MHz, $CDCl_3$) of (*R*)-aziridinyl diphenylmethanol *ent*-1.**

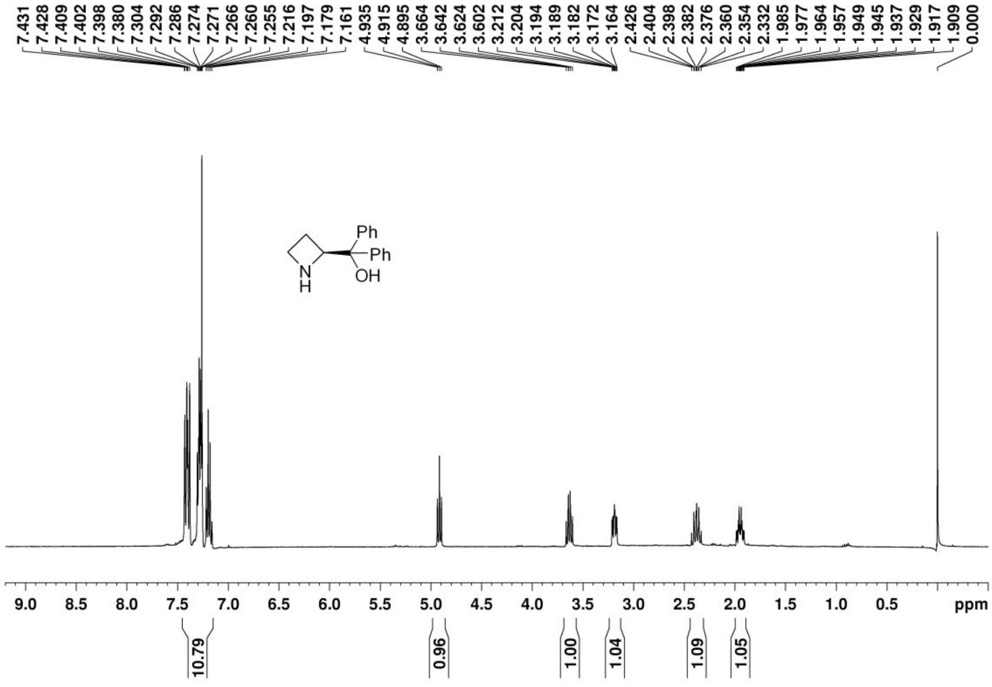

Figure S5. **^{1}H NMR (400 MHz, $CDCl_3$) of (*S*)-azetidinyl diphenylmethanol 2.**

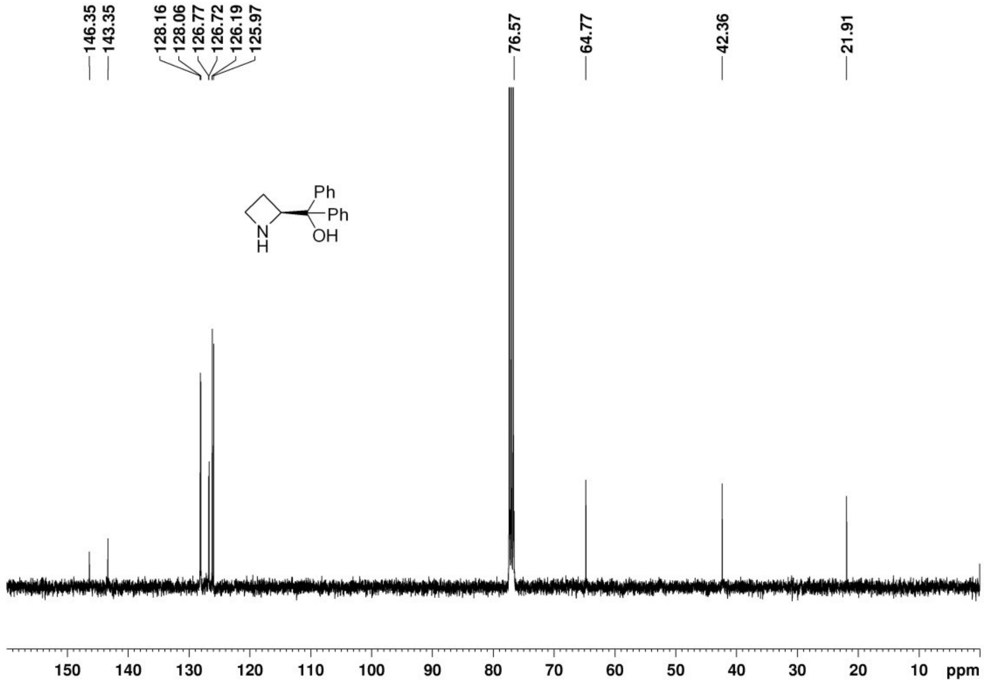

Figure S6. **^{13}C NMR (100 MHz, $CDCl_3$) of (*S*)-azetidinyl diphenylmethanol 2.**

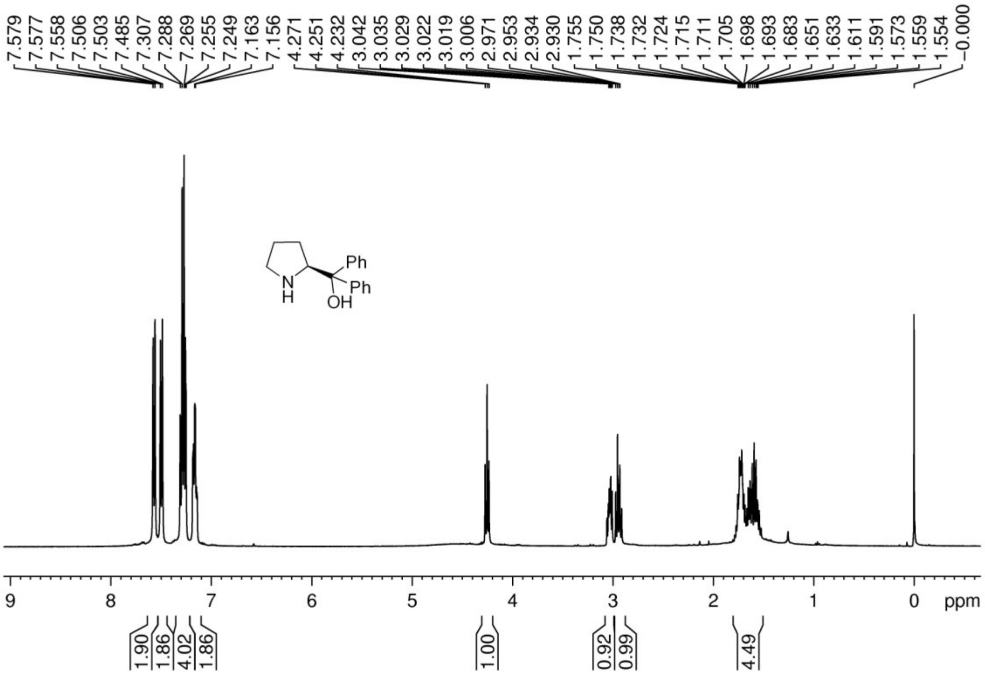

Figure S7. ^{1}H NMR (400 MHz, $CDCl_3$) of (*S*)-pyrrolidinyl diphenylmethanol 3.

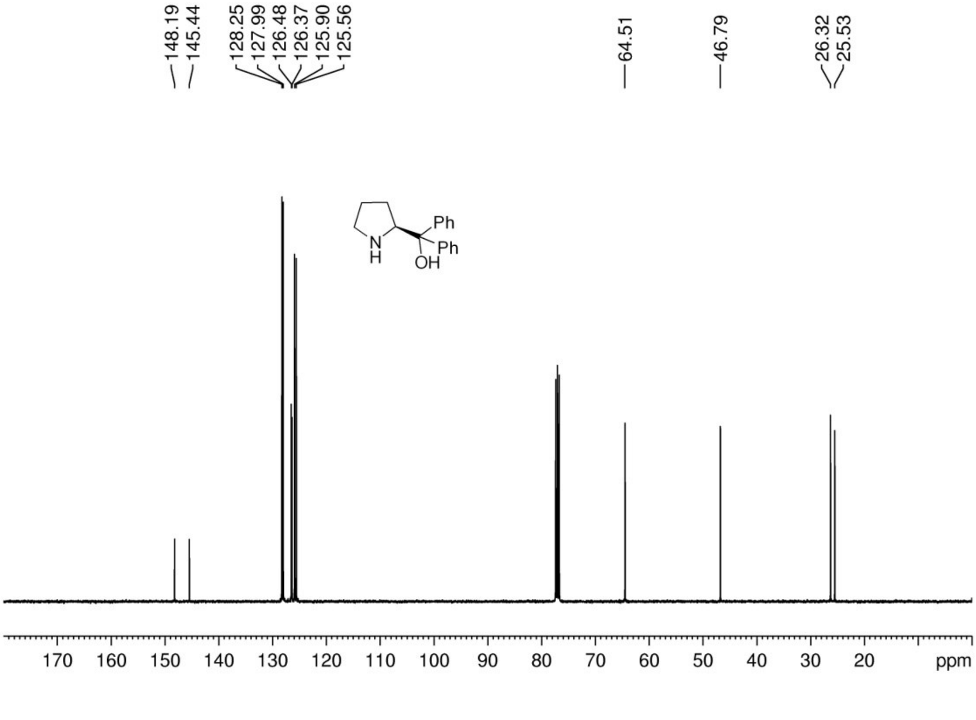

Figure S8. ^{13}C NMR (100 MHz, $CDCl_3$) of (*S*)-pyrrolidinyl diphenylmethanol 3.

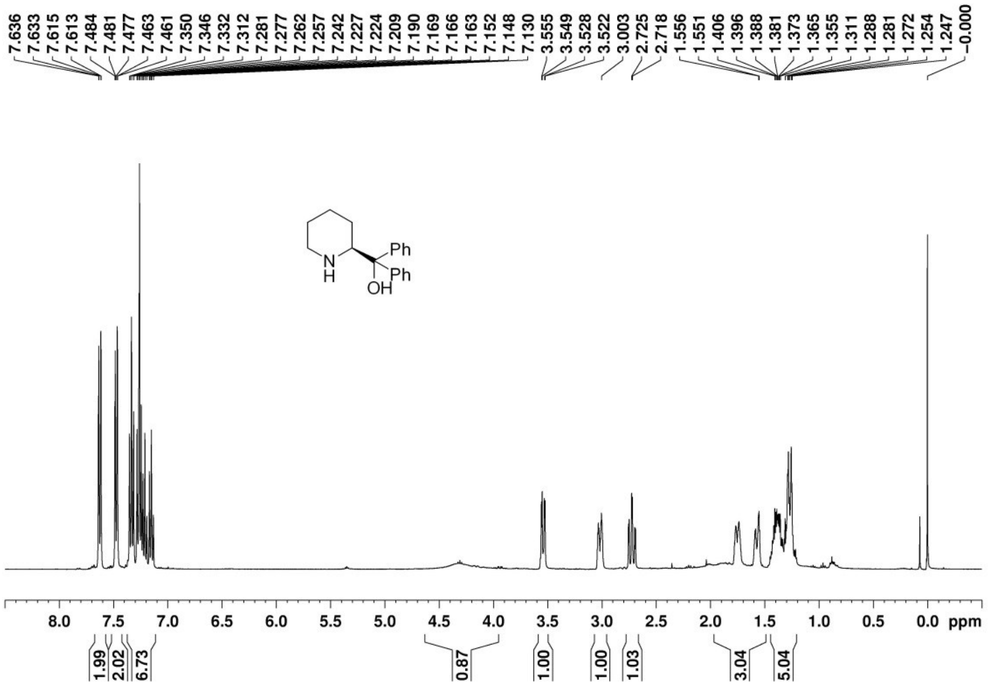

Figure S9. ^{1}H NMR (400 MHz, $CDCl_3$) of (*S*)-piperidinyl diphenylmethanol 4.

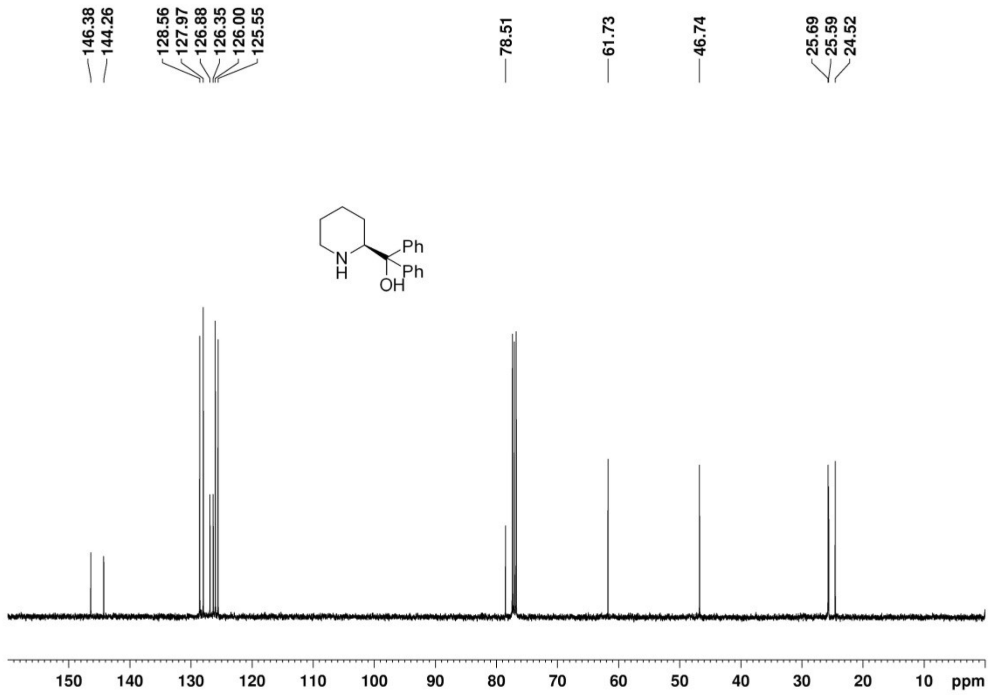

Figure S10. ^{13}C NMR (100 MHz, $CDCl_3$) of (*S*)-piperidinyl diphenylmethanol 4.

6. ^{1}H NMR spectroscopy CSA 1-4 and racemic 3,5-difluoro-mandelic acid

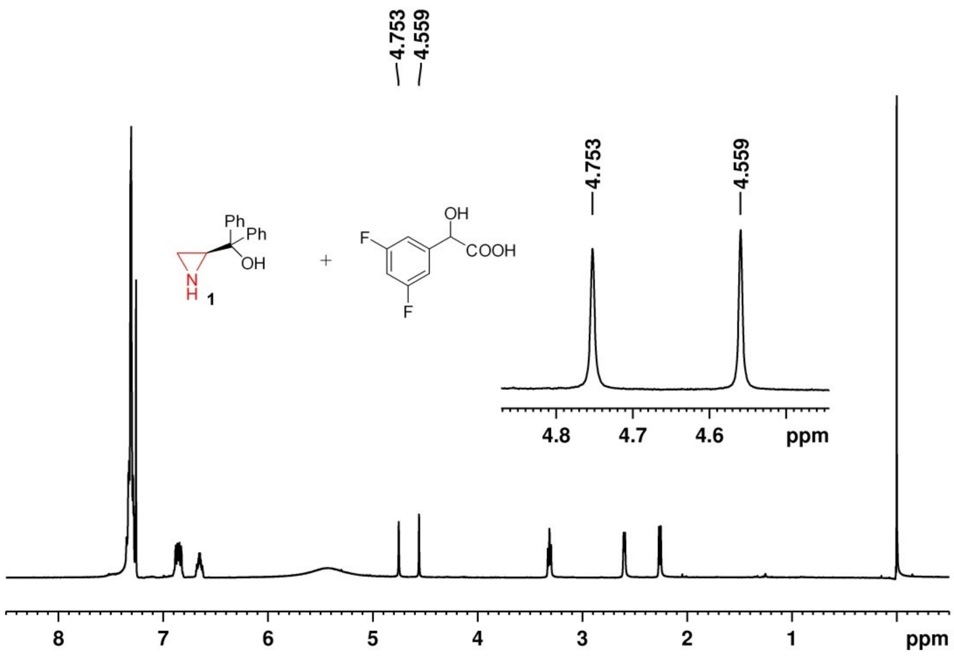

Figure S11. ^{1}H NMR Spectra (400 MHz, $CDCl_3$) of (*S*)-aziridinyl diphenylmethanol 1 and (±)-3,5-difluoro-mandelic acid.

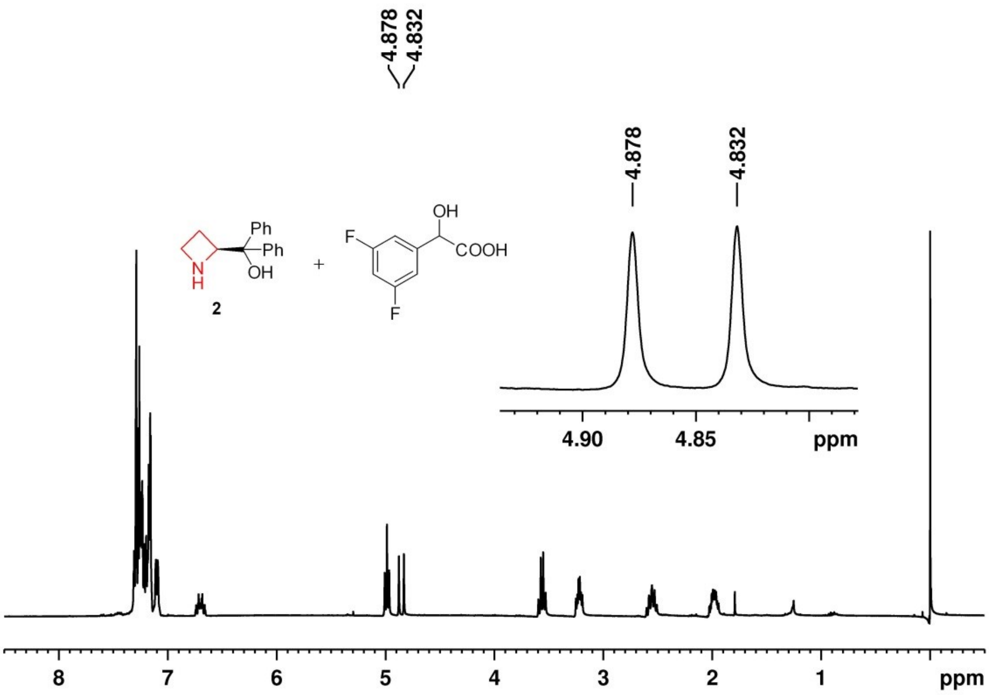

Figure S12. ^{1}H NMR Spectra (400 MHz, $CDCl_3$) of (*S*)-azetidinyl diphenylmethanol 2 and (±)-3,5-difluoro-mandelic acid.

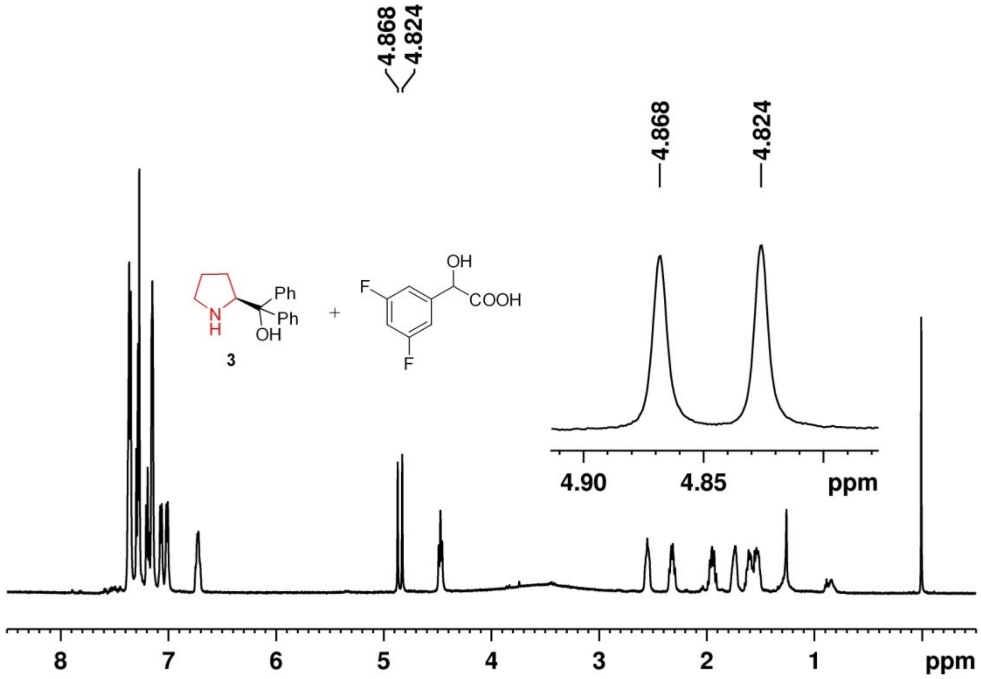

Figure S13. **^{1}H NMR Spectra (400 MHz, $CDCl_3$) of (*S*)-pyrrolidinyl diphenylmethanol 1 and (±)-3,5-difluoro-mandelic acid.**

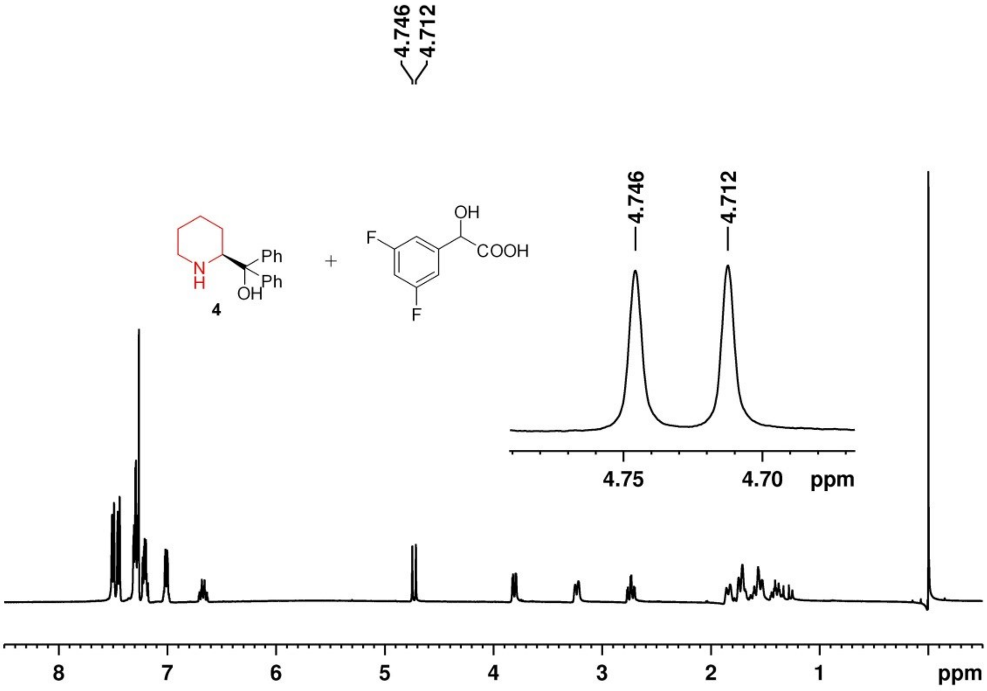

Figure S14. **^{1}H NMR Spectra (400 MHz, $CDCl_3$) of (*S*)-piperidinyl diphenylmethanol 4 and (±)-3,5-difluoro-mandelic acid.**

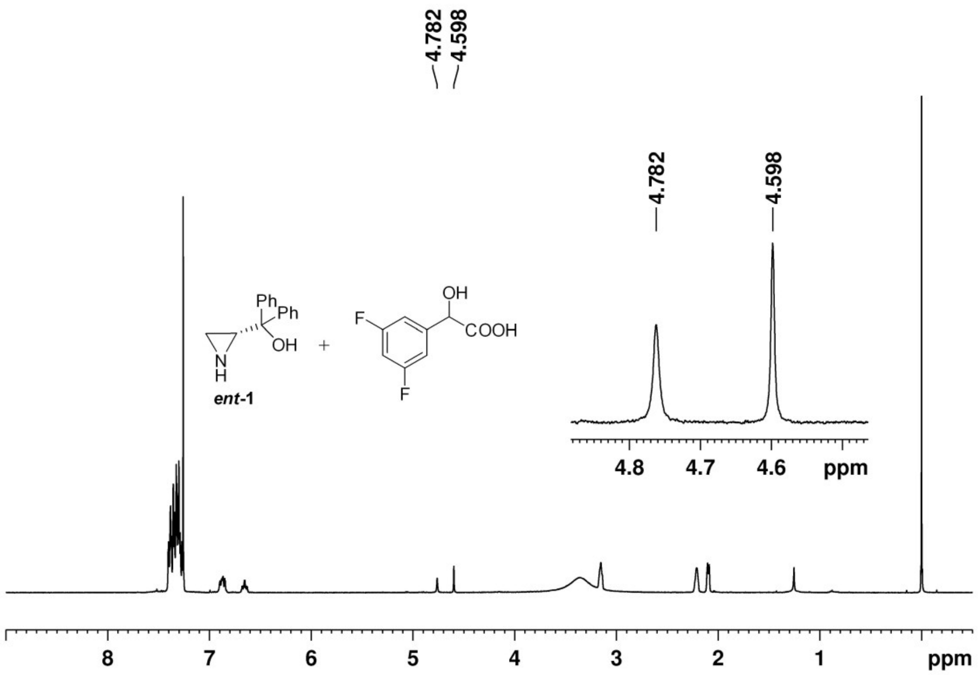

Figure S15. **^{1}H NMR Spectra (400 MHz, $CDCl_3$) of (*R*)-aziridinyl diphenylmethanol *ent*-1 and (±)-3,5-difluoro-mandelic acid.**

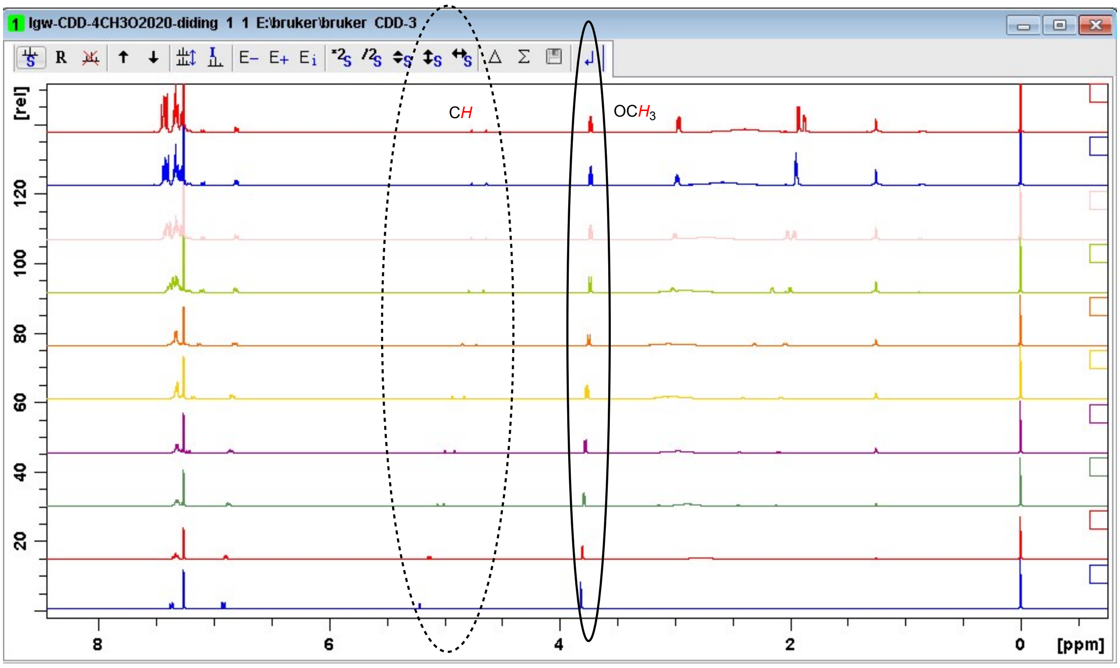

Figure S16. Evolution of ^{1}H NMR (400 MHz, $CDCl_3$) signals of methine and methoxy group of (*rac*)-4-MeO-MA by NMR titration experiments.

7. ^{1}H NMR spectroscopy (*S*)-aziridinyl diphenylmethanol and various racemic α-substituted carboxylic acids

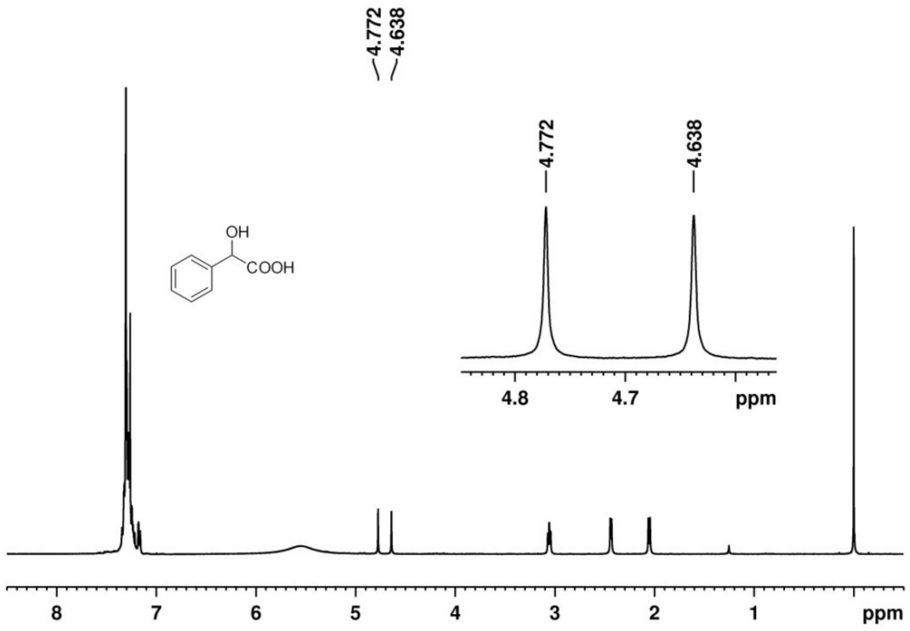

Figure S1a. ^{1}H NMR Spectra (400 MHz, $CDCl_3$) of (*S*)-aziridinyl diphenylmethanol 1 and (±)-mandelic acid.

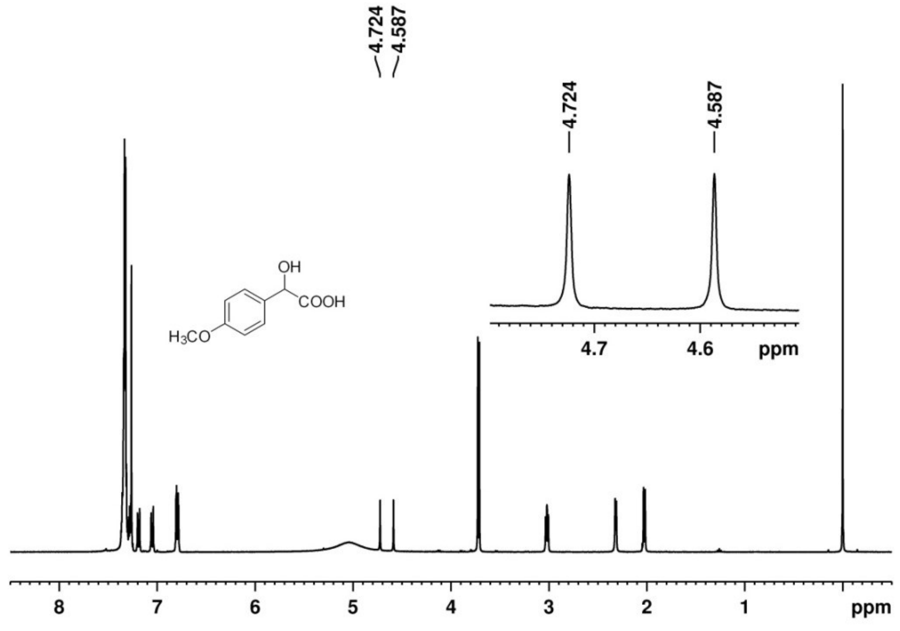

Figure S1b. ^{1}H NMR Spectra (400 MHz, $CDCl_3$) of (*S*)-aziridinyl diphenylmethanol 1 and (±)-4-methoxy-mandelic acid.

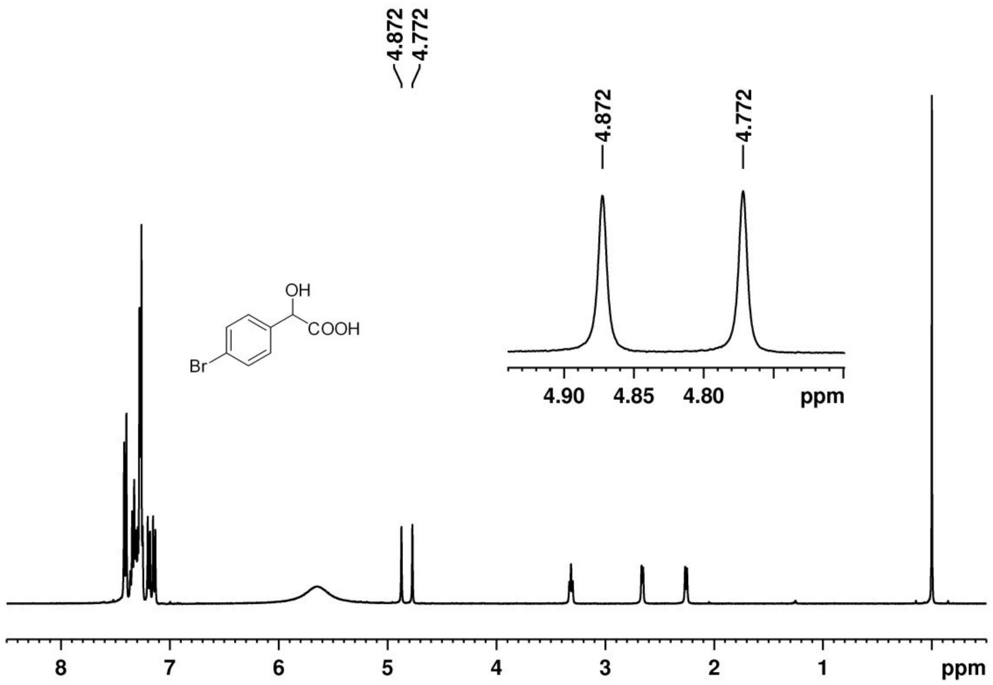

Figure S1c. **^{1}H NMR Spectra (400 MHz, $CDCl_3$) of (*S*)-aziridinyl diphenylmethanol 1 and (±)-4-bromo-mandelic acid.**

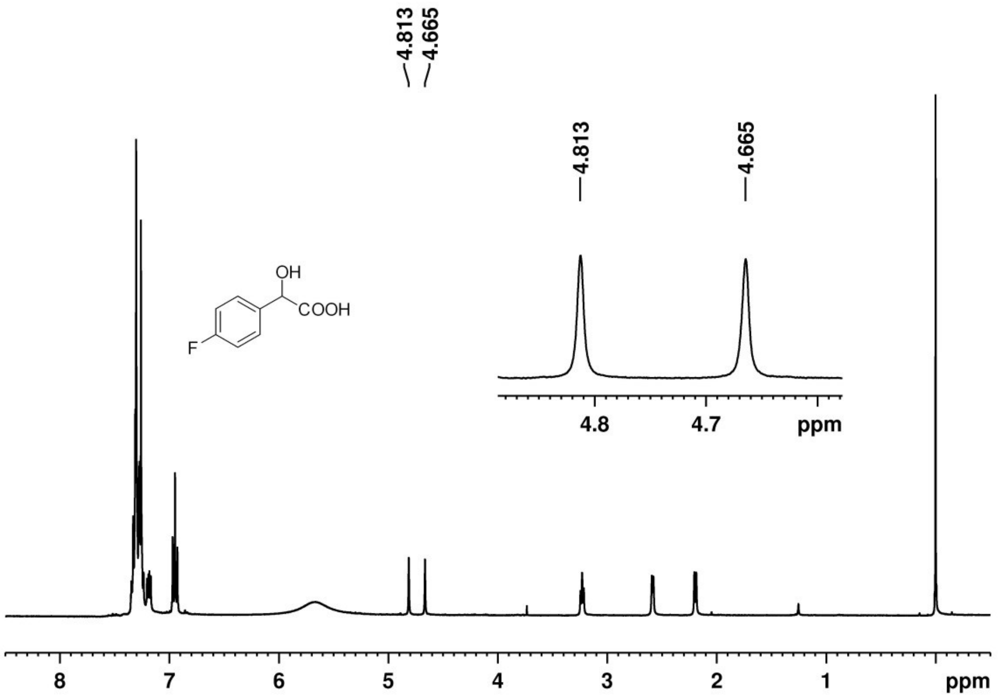

Figure S1d. **^{1}H NMR Spectra (400 MHz, $CDCl_3$) of (*S*)-aziridinyl diphenylmethanol 1 and (±)-4-fluoro-mandelic acid.**

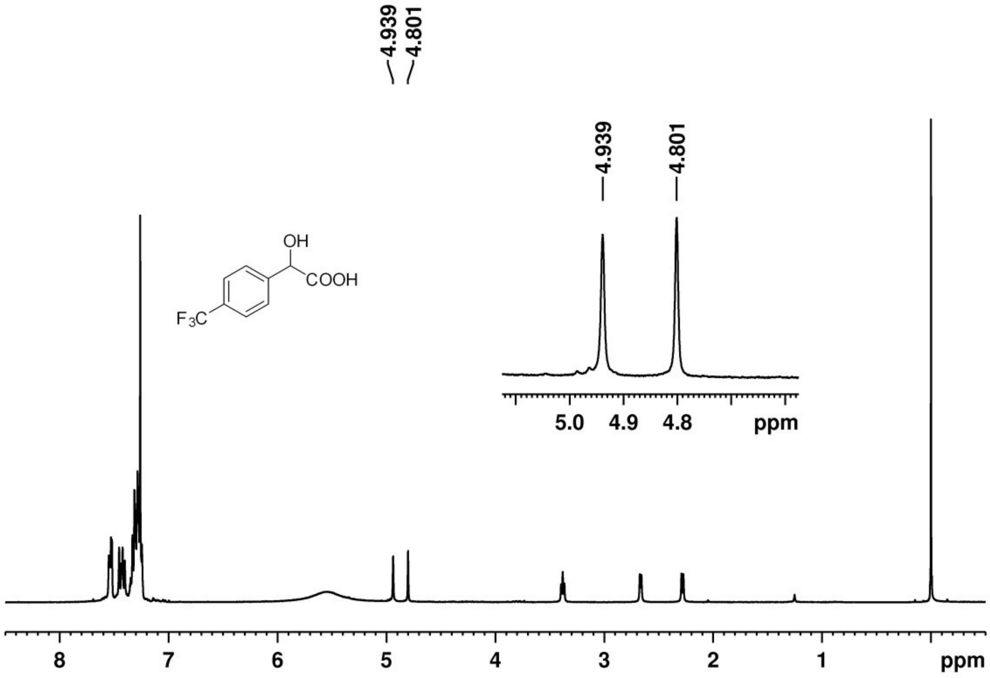

Figure S1e. **^{1}H NMR Spectra (400 MHz, $CDCl_3$) of (*S*)-aziridinyl diphenylmethanol 1 and (±)-4-trifluoromethyl-mandelic acid.**

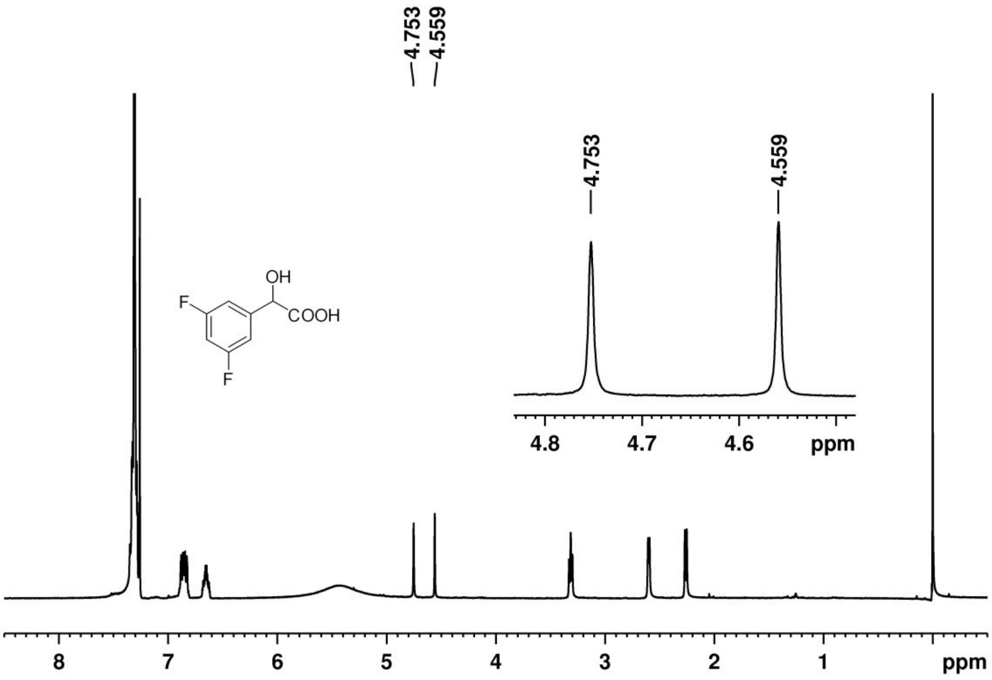

Figure S1f. **^{1}H NMR Spectra (400 MHz, $CDCl_3$) of (*S*)-aziridinyl diphenylmethanol 1 and (±)-3,5-difluoro-mandelic acid.**

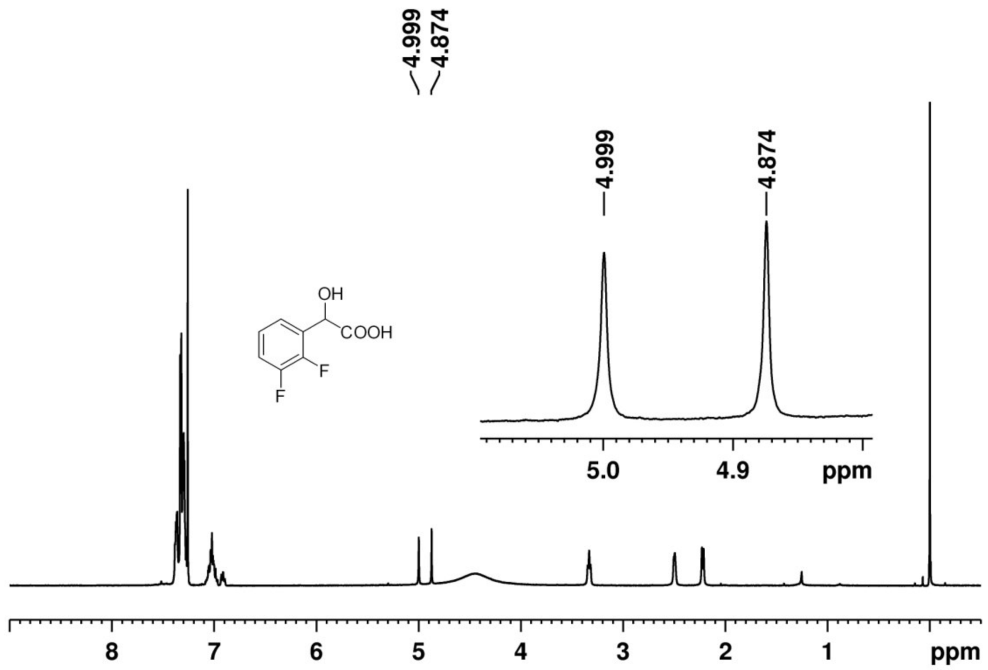

Figure S1g. **^{1}H NMR Spectra (400 MHz, $CDCl_3$) of (*S*)-aziridinyl diphenylmethanol 1 and (±)-2,3-difluoro-mandelic acid.**

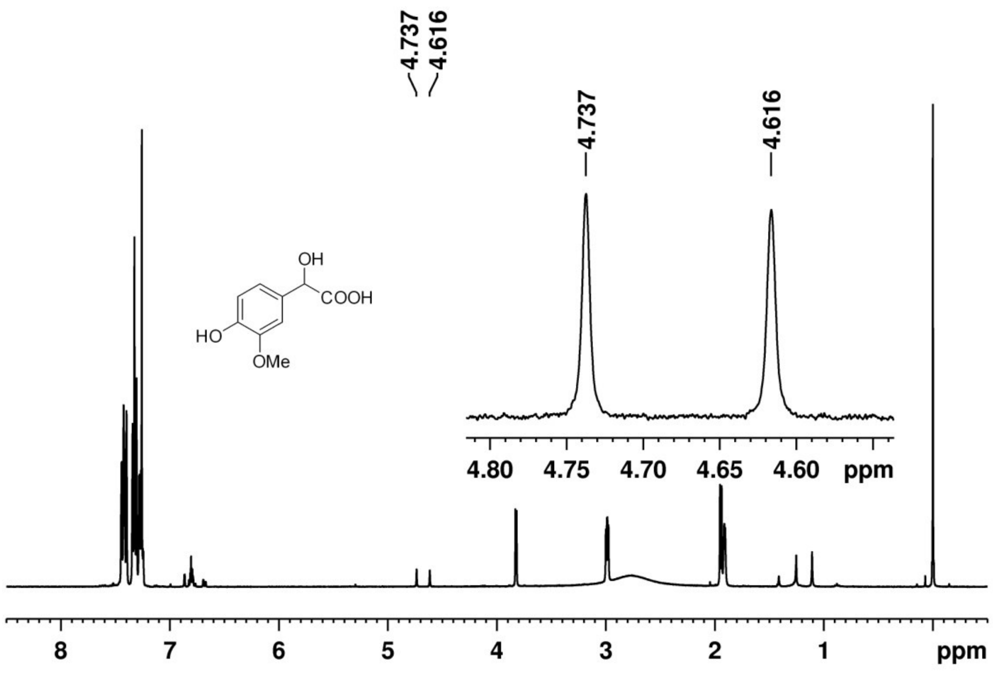

Figure S1h. **^{1}H NMR Spectra (400 MHz, $CDCl_3$) of (*S*)-aziridinyl diphenylmethanol 1 and (±)-4-hydroxy-3-methoxy-mandelic acid.**

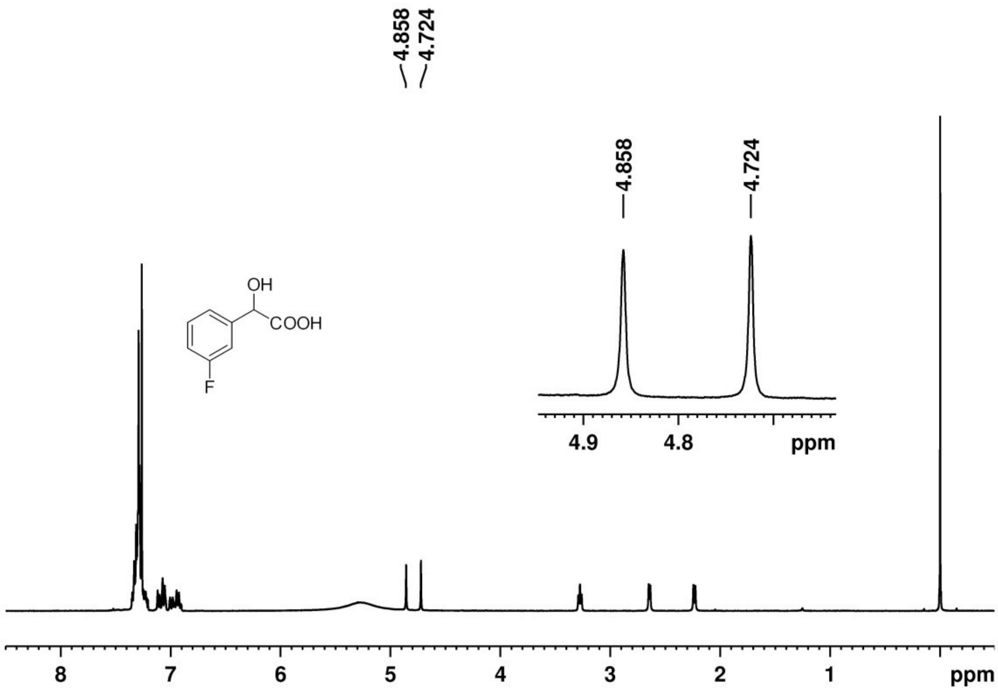

Figure S1i. **^{1}H NMR Spectra (400 MHz, $CDCl_3$) of (*S*)-aziridinyl diphenylmethanol 1 and (±)-3-fluoro-mandelic acid.**

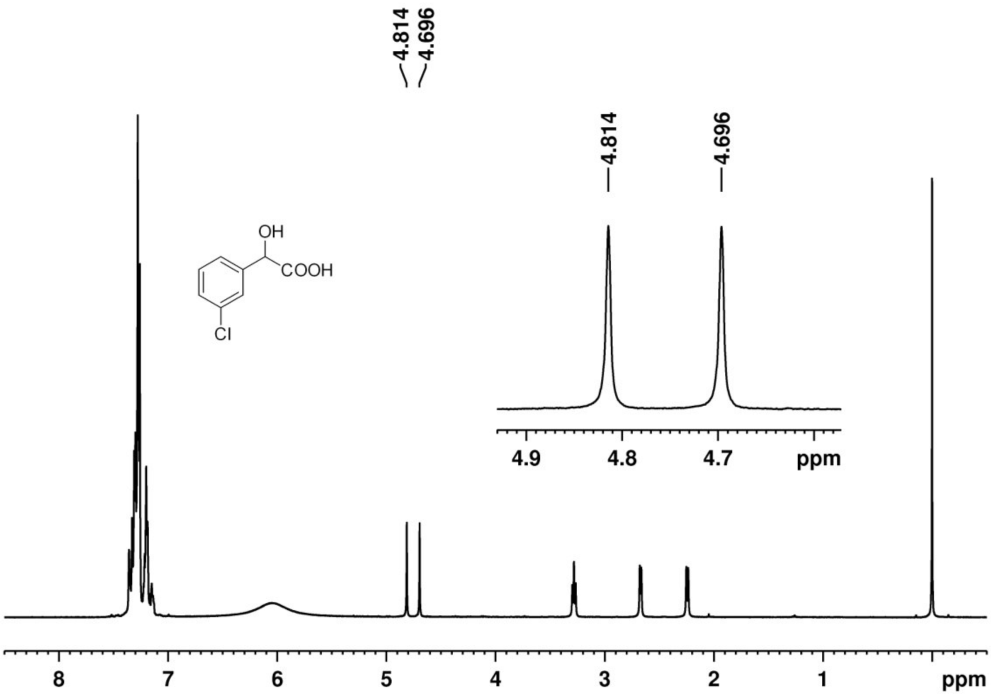

Figure S1j. **^{1}H NMR Spectra (400 MHz, $CDCl_3$) of (*S*)-aziridinyl diphenylmethanol 1 and (±)-3-chloro-mandelic acid.**

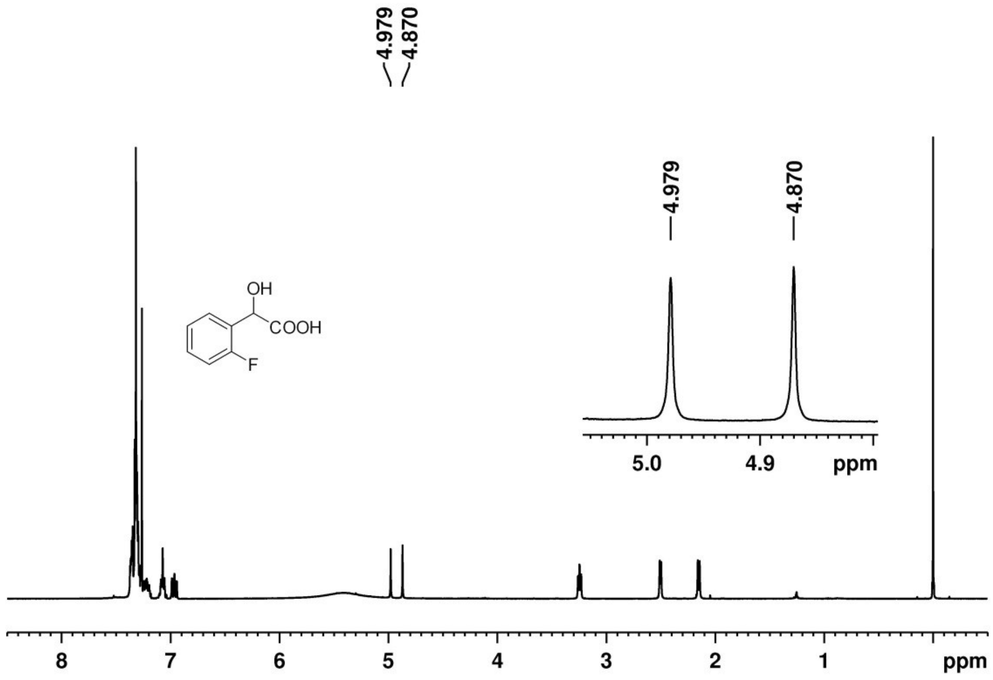

Figure S1k. **^{1}H NMR Spectra (400 MHz, $CDCl_3$) of (*S*)-aziridinyl diphenylmethanol 1 and (±)-2-fluoro-mandelic acid.**

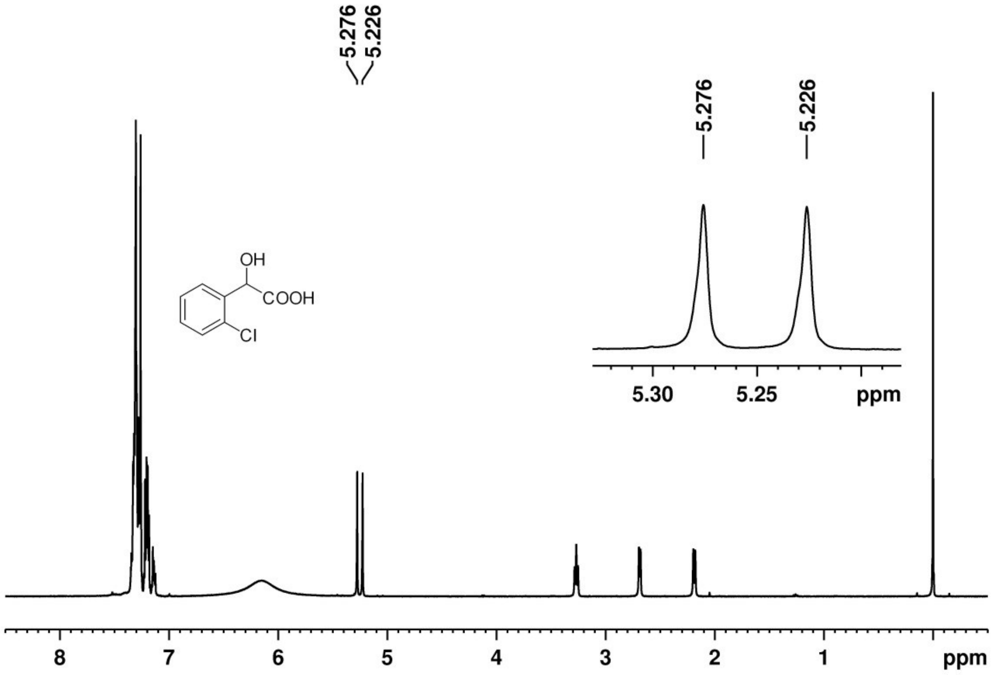

Figure S1l. **^{1}H NMR Spectra (400 MHz, $CDCl_3$) of (*S*)-aziridinyl diphenylmethanol 1 and (±)-2-chloro-mandelic acid.**

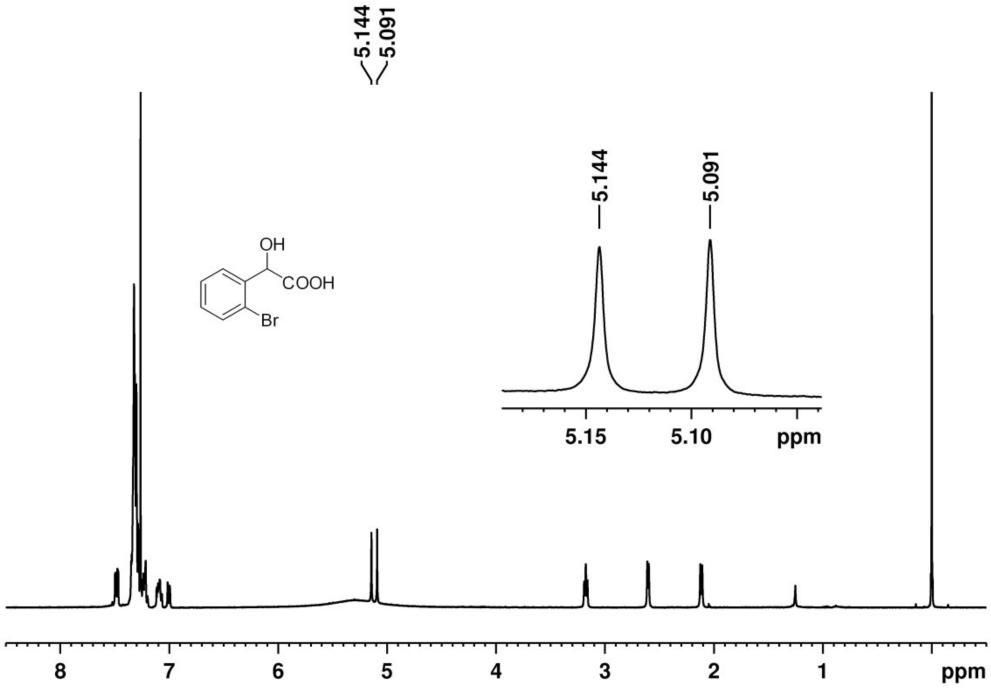

Figure S1m. ^{1}H NMR Spectra (400 MHz, $CDCl_3$) of (*S*)-aziridinyl diphenylmethanol 1 and (±)-2-bromo-mandelic acid.

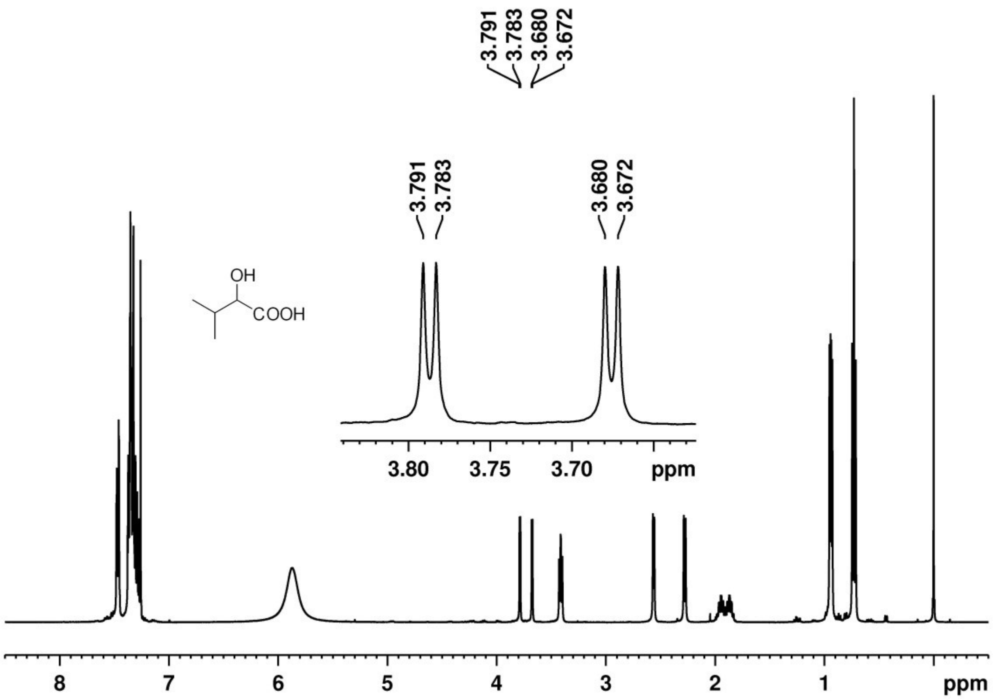

Figure Sln. ^{1}H NMR Spectra (400 MHz, $CDCl_3$) of (*S*)-aziridinyl diphenylmethanol 1 and (±)-2-hydroxy-3-methylbutyric acid.

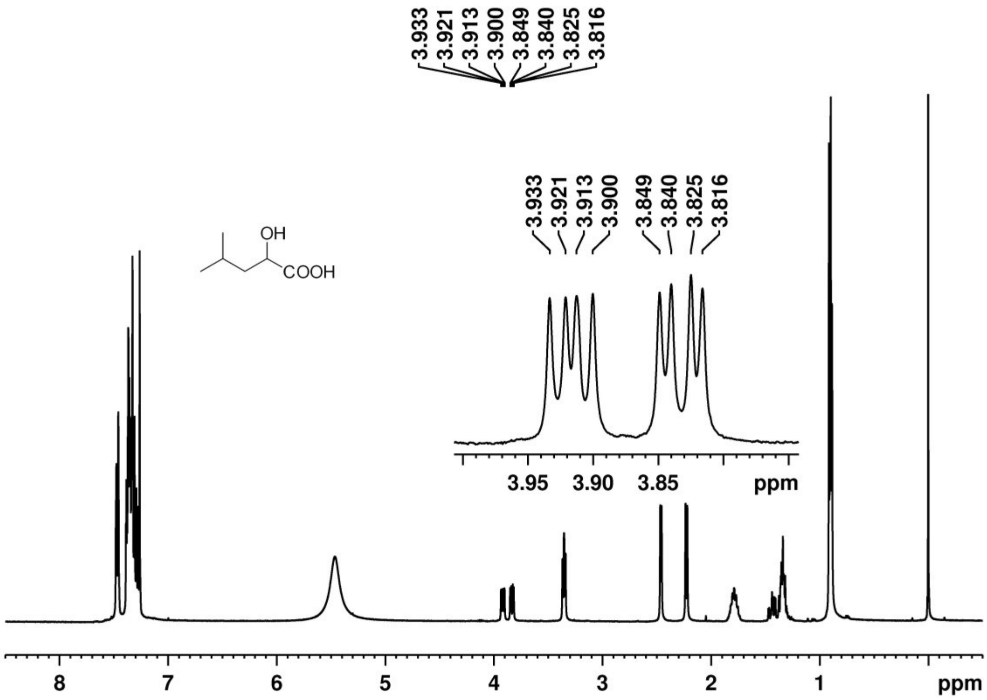

Figure S1o. **^{1}H NMR Spectra (400 MHz, $CDCl_3$) of (*S*)-aziridinyl diphenylmethanol 1 and (±)-2-hydroxyisocaproic acid.**

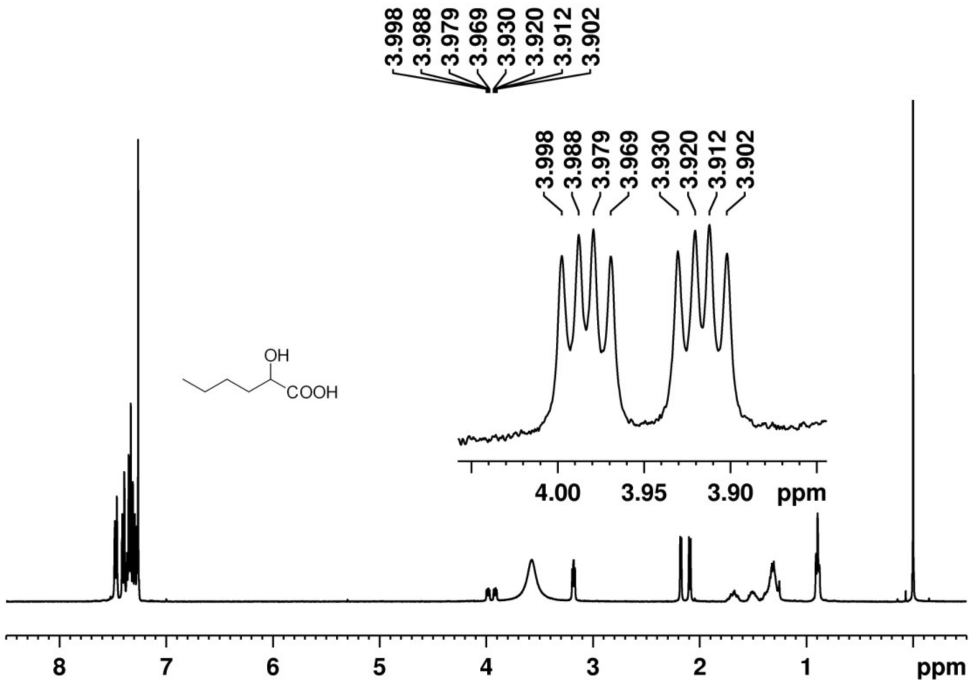

Figure S1p. **^{1}H NMR Spectra (400 MHz, $CDCl_3$) of (*S*)-aziridinyl diphenylmethanol 1 and (±)-2-hydroxyhexanoic acid.**

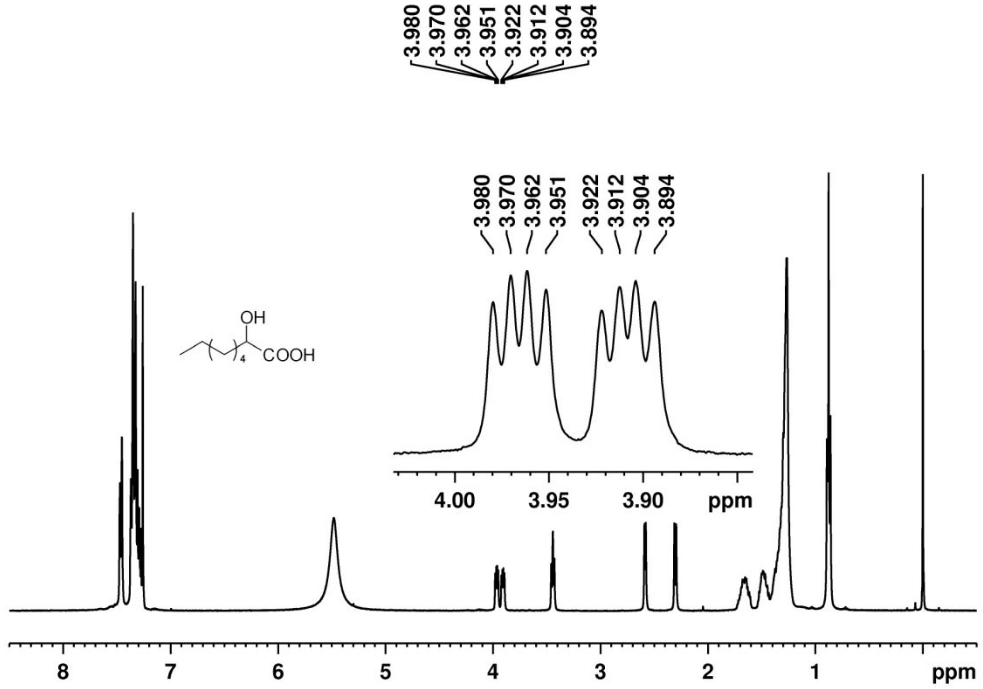

Figure S1q. ¹H NMR Spectra (400 MHz, CDCl₃) of (*S*)-aziridinyl diphenylmethanol 1 and (±)-2-hydroxyoctanoic acid.

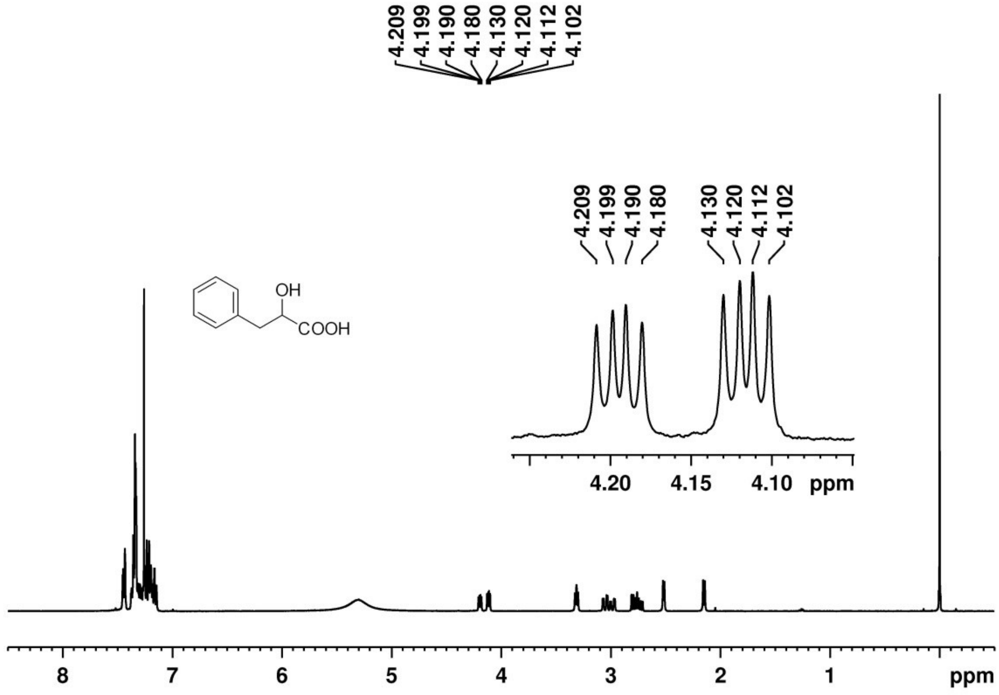

Figure S1r. ¹H NMR Spectra (400 MHz, CDCl₃) of (*S*)-aziridinyl diphenylmethanol 1 and (±)-2-hydroxy-3-phenylpropanoic acid.

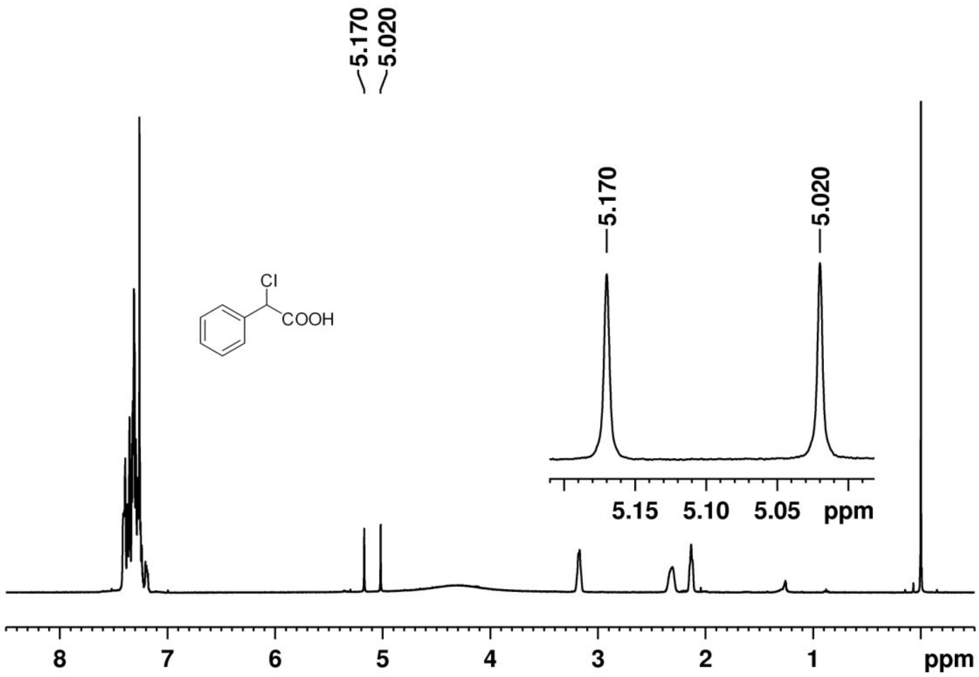

Figure S1s. ^{1}H NMR Spectra (400 MHz, $CDCl_3$) of (*S*)-aziridinyl diphenylmethanol 1 and (±)-α-chloro-phenylacetic acid .

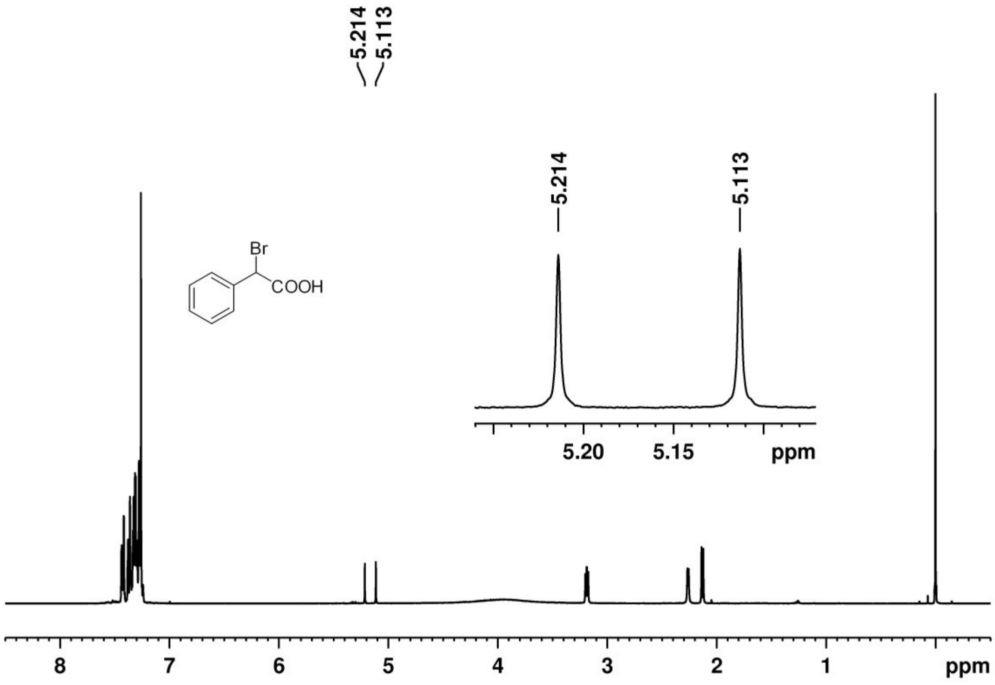

Figure S1t. ^{1}H NMR Spectra (400 MHz, $CDCl_3$) of (*S*)-aziridinyl diphenylmethanol 1 and (±)-α-bromo-phenylacetic acid.

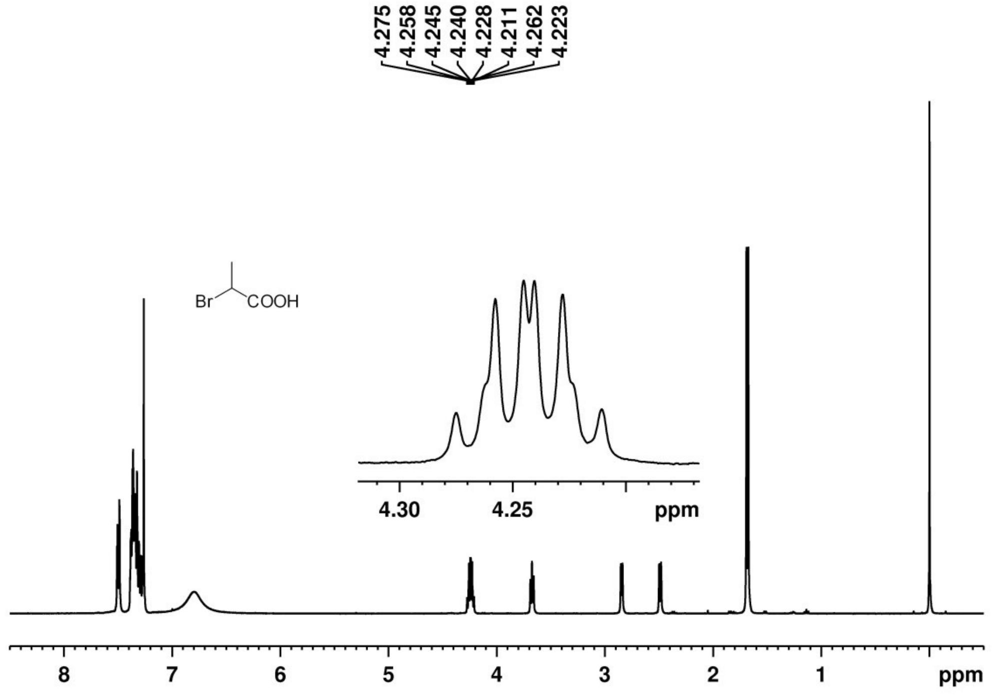

Figure S1u. **^{1}H NMR Spectra (400 MHz, $CDCl_3$) of (*S*)-aziridinyl diphenylmethanol 1 and (±)-2-bromopropanoic acid.**

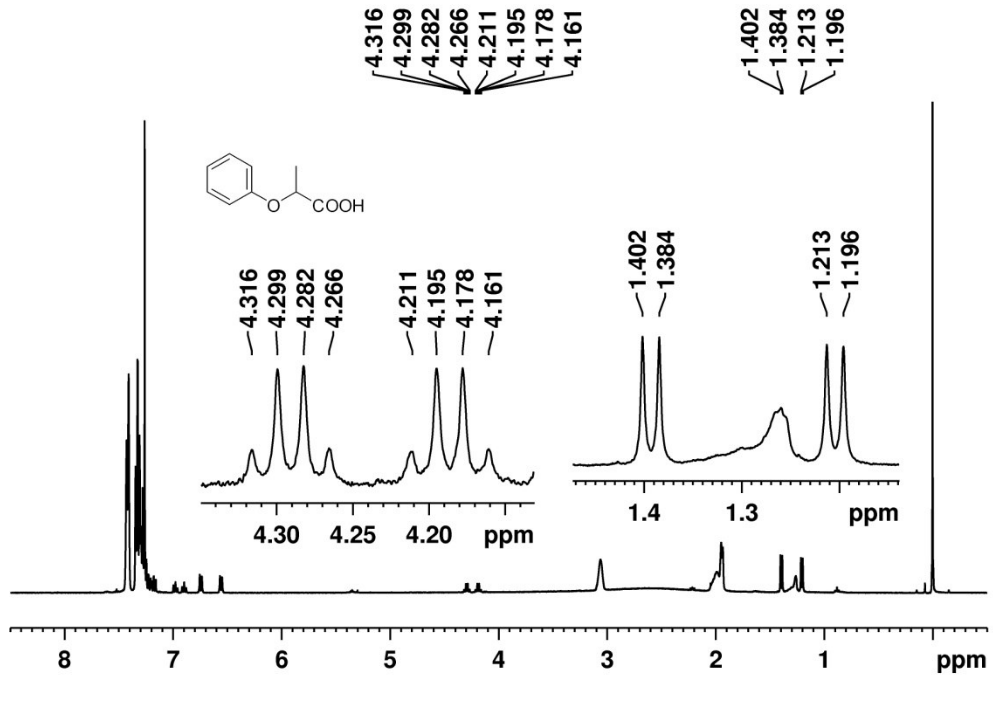

Figure S1v. **^{1}H NMR Spectra (400 MHz, $CDCl_3$) of (*S*)-aziridinyl diphenylmethanol 1 and (±)-2-phenoxypropanoic acid.**

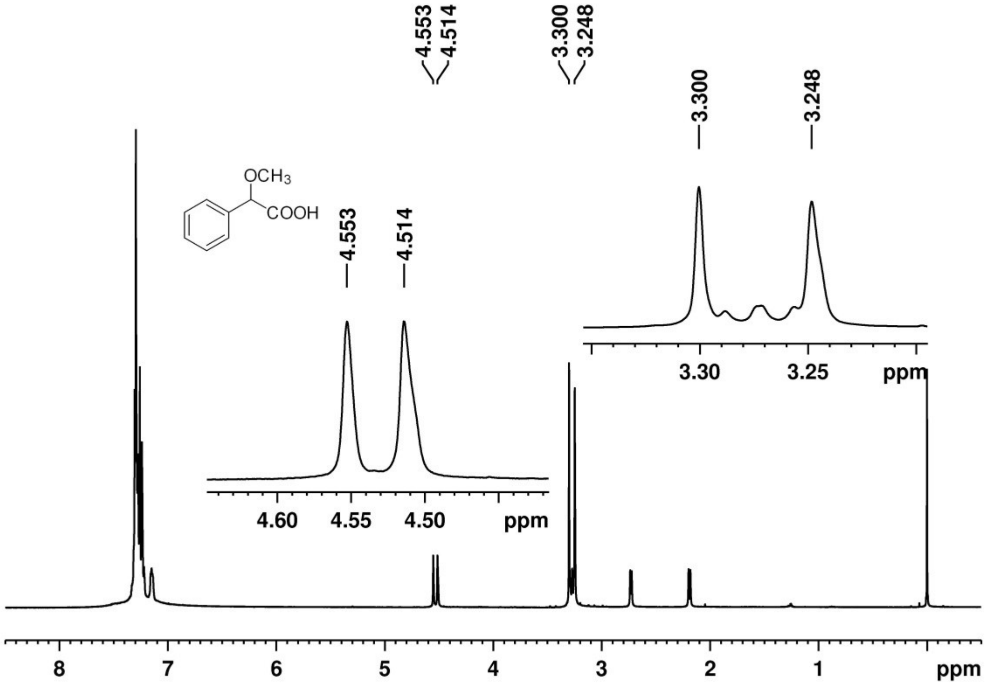

Figure S1w. ^{1}H NMR Spectra (400 MHz, $CDCl_3$) of (*S*)-aziridinyl diphenylmethanol 1 and (±)-2-methoxy-2-phenylacetic acid.

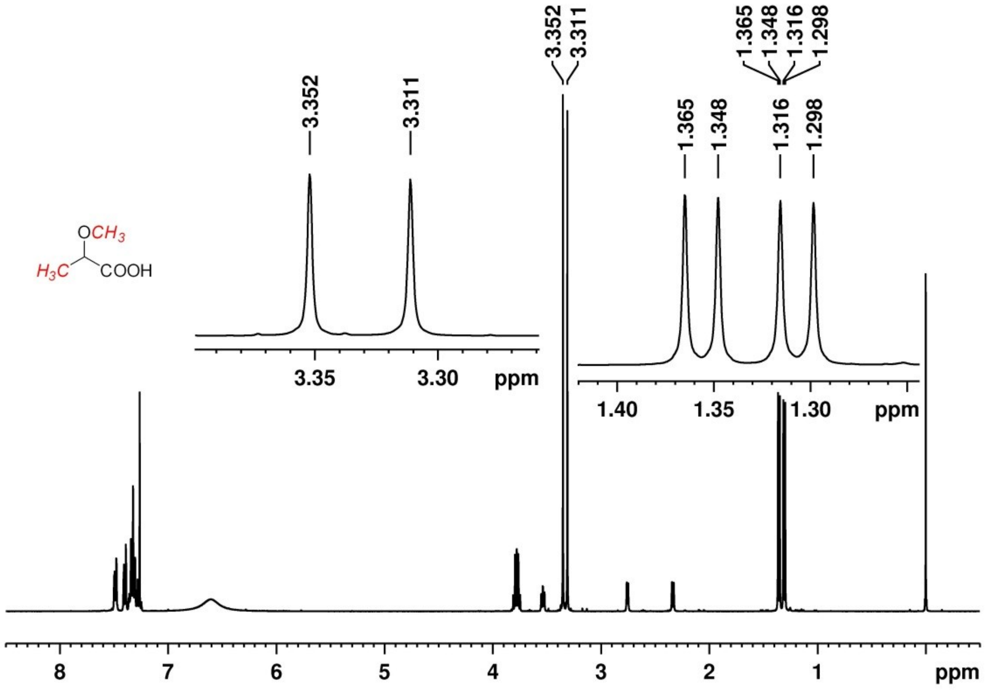

Figure S1x. ^{1}H NMR Spectra (400 MHz, $CDCl_3$) of (*S*)-aziridinyl diphenylmethanol 1 and (±)-2-methoxypropanoic acid.

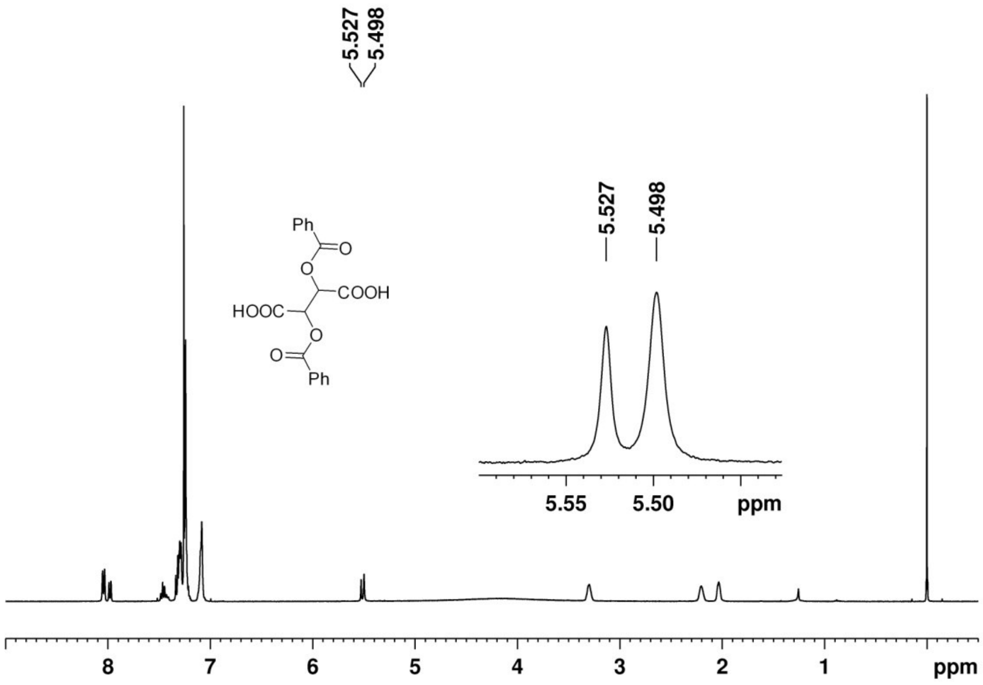

Figure S1y. ^{1}H NMR Spectra (400 MHz, $CDCl_3$) of (*S*)-aziridinyl diphenylmethanol 1 and (±)-2,3-bis(benzoyloxy)succinic acid.

8. ^{19}F NMR spectroscopy (*S*)-aziridinyl diphenylmethanol and fluorine-containing α-substituted carboxylic acids

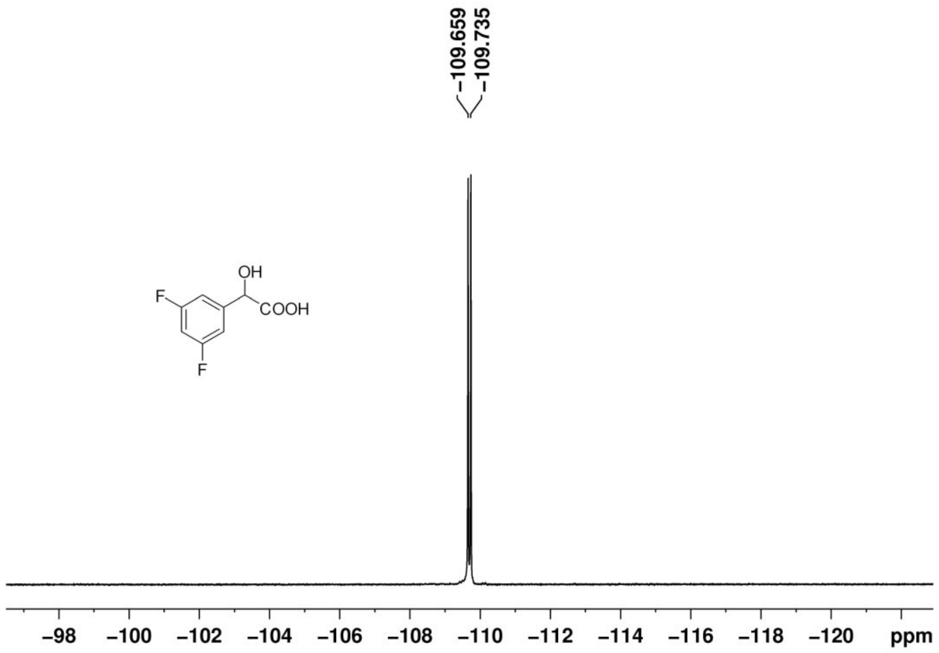

Figure S2a. ^{19}F NMR Spectra (376 MHz, $CDCl_3$) of (*S*)-aziridinyl diphenylmethanol 1 and (±)-3,5-difluoro-mandelic acid

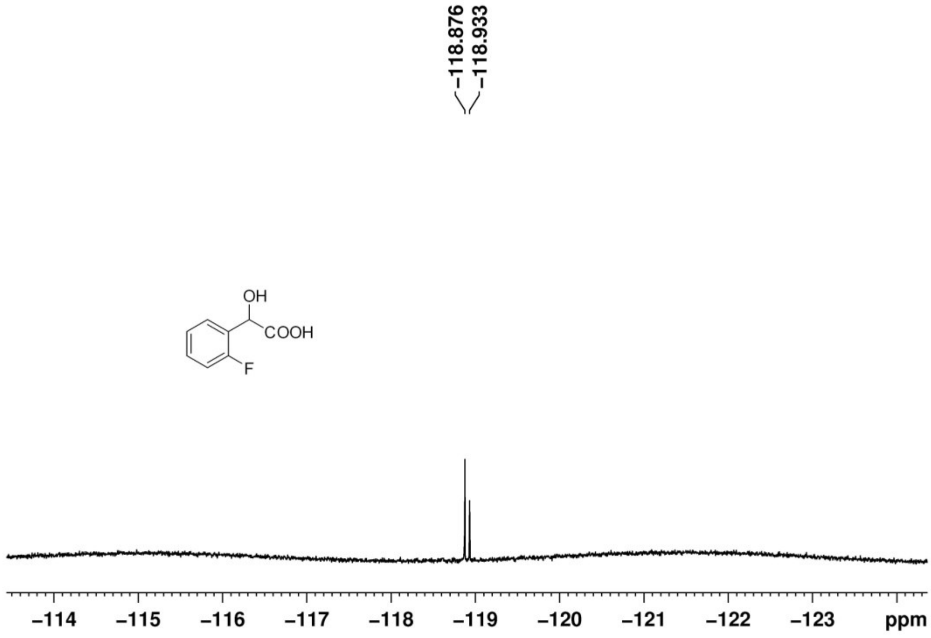

Figure S2b. ^{19}F NMR Spectra (376 MHz, $CDCl_3$) of (*S*)-aziridinyl diphenylmethanol 1 and (±)-2-fluoro-mandelic acid

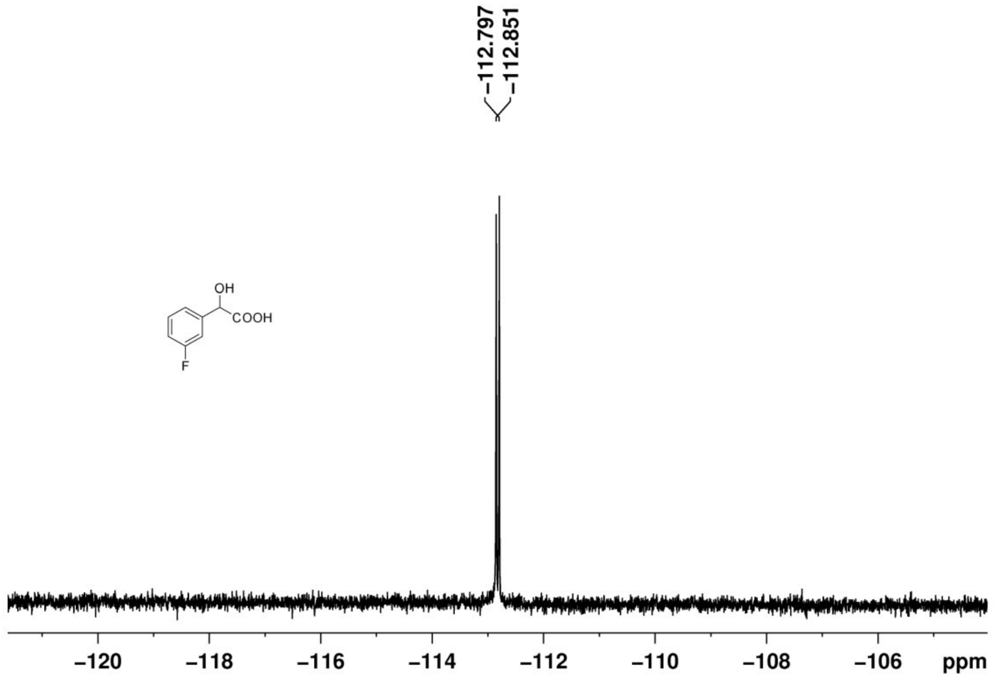

Figure S2c. ^{19}F **NMR Spectra (376 MHz,** $CDCl_3$**) of (*S*)-aziridinyl diphenylmethanol 1 and (±)-3-fluoro-mandelic acid**

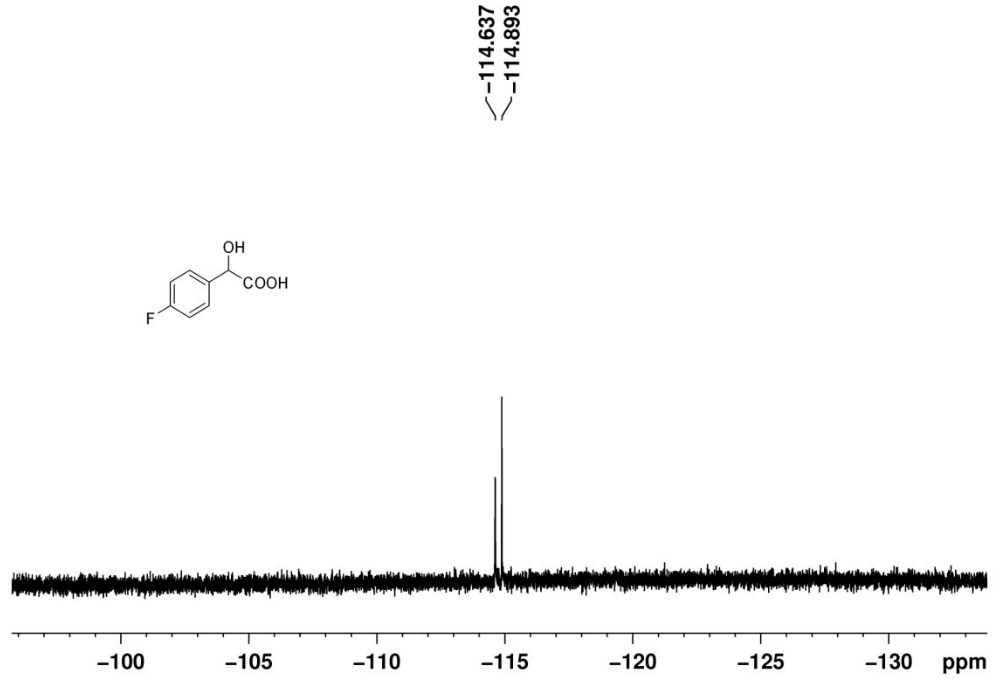

Figure S2d. ^{19}F **NMR Spectra (376 MHz,** $CDCl_3$**) of (*S*)-aziridinyl diphenylmethanol 1 and (±)-4-fluoro-mandelic acid**

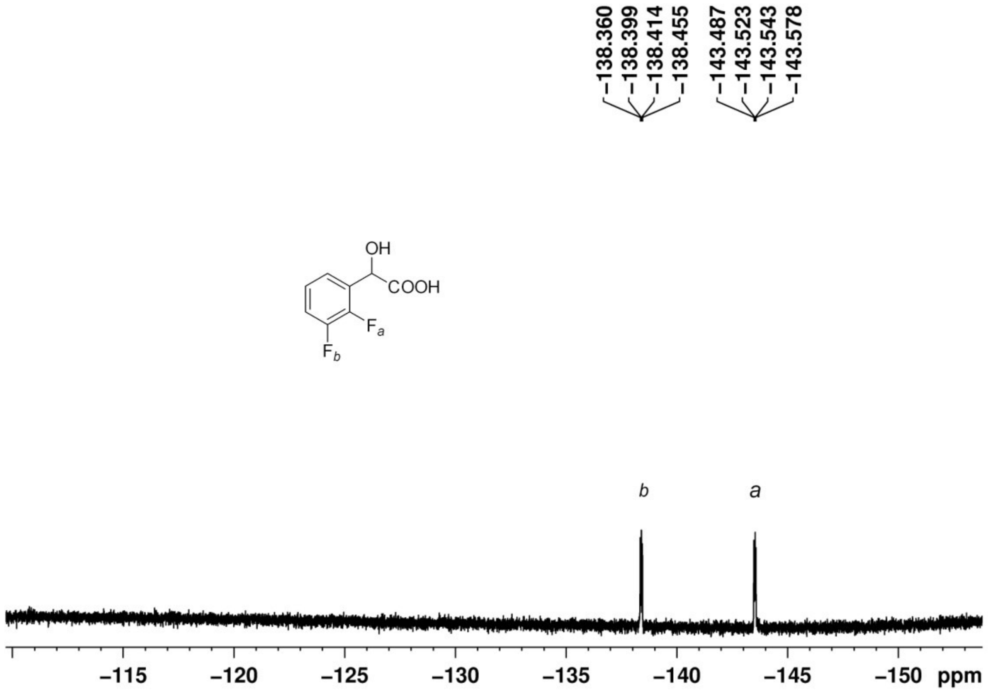

Figure S2e. ^{19}F NMR Spectra (376 MHz, $CDCl_3$) of (*S*)-aziridinyl diphenylmethanol 1 and (±)-2,3-difluoro-mandelic acid

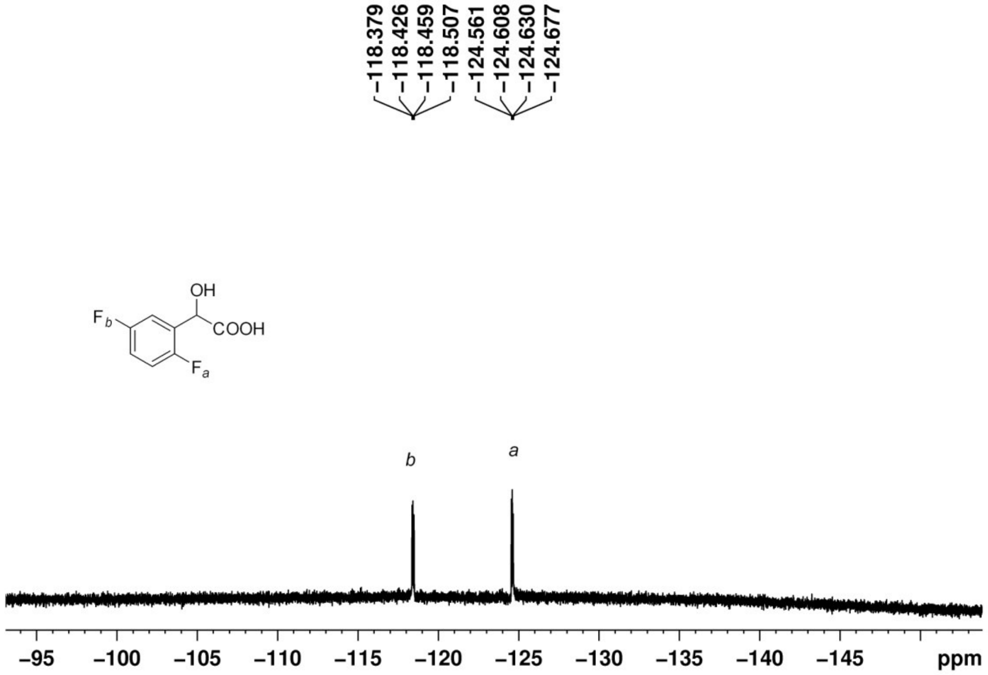

Figure S2f. ^{19}F NMR Spectra (376 MHz, $CDCl_3$) of (*S*)-aziridinyl diphenylmethanol 1 and (±)-2,5-difluoro-mandelic acid

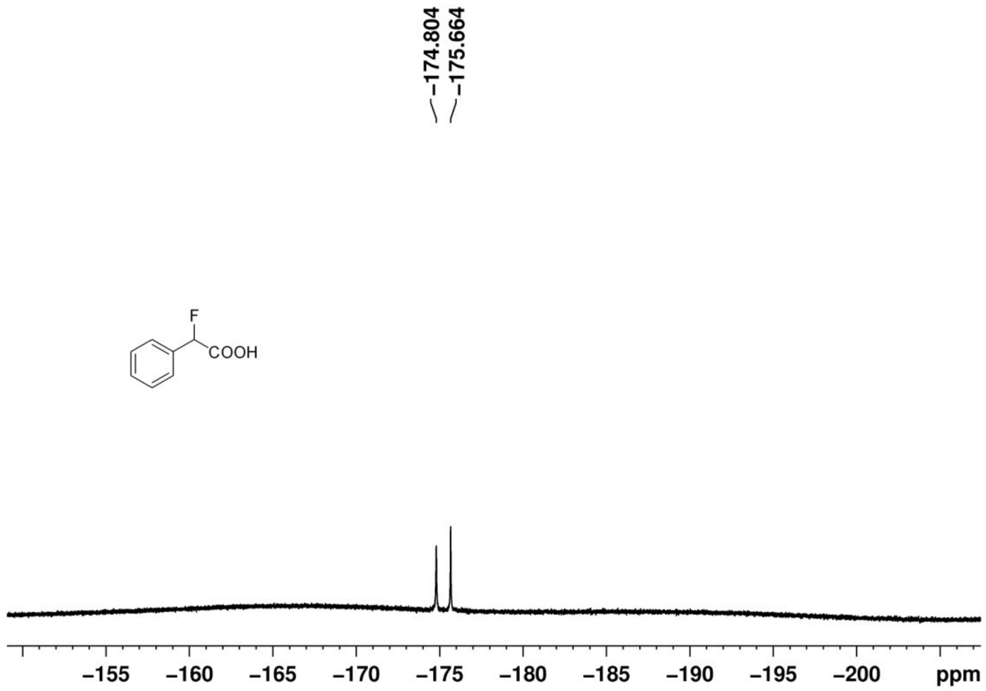

Figure S2g. ^{19}**F NMR Spectra (376 MHz, $CDCl_3$) of (*S*)-aziridinyl diphenylmethanol 1 and (±)-α-fluoro-phenylacetic acid.**

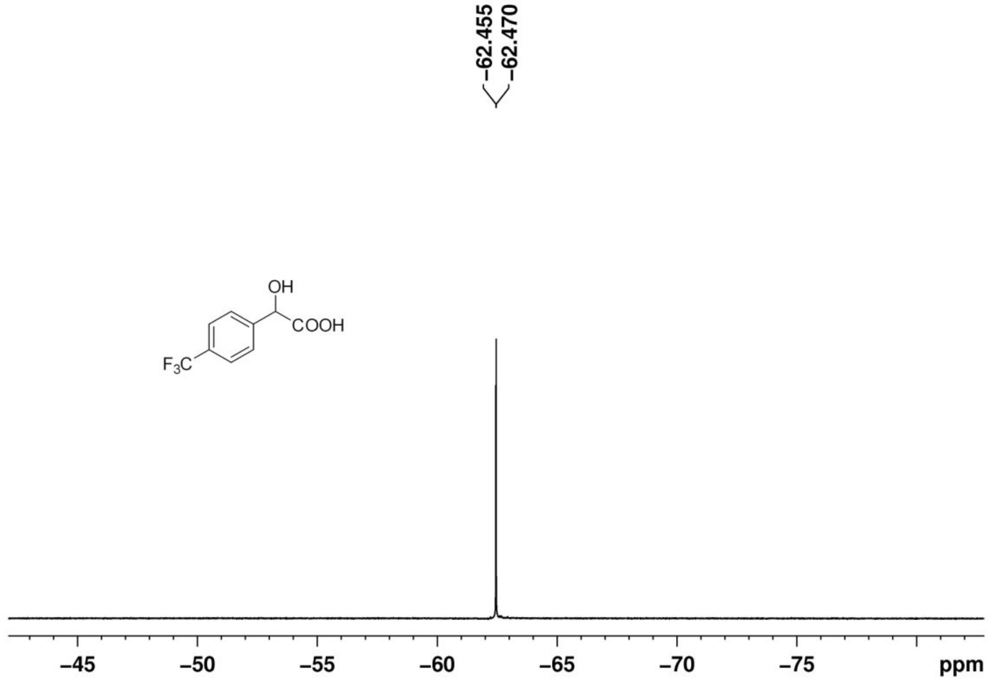

Figure S2h. ^{19}**F NMR Spectra (376 MHz, $CDCl_3$) of (*S*)-aziridinyl diphenylmethanol 1 and (±)-4-trifluoromethyl-mandelic acid**
